# Differential AXL expression and Arf1 regulation control stiffness-dependent Golgi organization in breast cancer cells

**DOI:** 10.1242/jcs.263956

**Published:** 2026-02-03

**Authors:** Arnav Saha, Tushar Sherkhane, Nagaraj Balasubramanian

**Affiliations:** IISER Pune, Dr. Homi Bhabha Road, Pune 411008, India

**Keywords:** Adhesion, Mechanosensing, Matrix stiffness, Golgi organization, AXL, Arf1, Breast cancer

## Abstract

Integrin-mediated adhesion regulates cellular survival and mechanotransduction, processes often deregulated in cancers. During breast tumor progression, matrix stiffening influences cytoskeletal organization, although its effect on organelle organization and function remains unclear. Here, we examine how Golgi organization responds to matrix stiffness sensing in breast cancer cells. In adherent MDA-MB-231 cells, the Golgi becomes progressively more compact and organized with increasing matrix stiffness, accompanied by enhanced tubulin acetylation, indicating stiffness-dependent regulation. In contrast, MCF7 cells display a diffused or disorganized Golgi regardless of matrix stiffness. AXL, a receptor tyrosine kinase differentially expressed in MDA-MB-231 cells and absent in MCF7, localizes prominently to the Golgi. Inhibition or knockdown of AXL disrupted stiffness-dependent Golgi organization in MDA-MB-231 cells, whereas stable AXL expression in MCF7 restored Golgi organization at higher stiffness. A stiffness-dependent increase in AXL and Arf1 expression regulates Arf1 activation and localization to control mechanosensitive Golgi organization. Inhibition of AXL and/or Arf1 disrupted Golgi organization, tubulin acetylation and cell-surface glycosylation. Together, our findings reveal a mechanoresponsive AXL-Arf1-Golgi signaling axis that integrates matrix stiffness sensing with Golgi organization and function in breast cancer cells.

## Teaser

**Highlighted Article:** An AXL-Arf1-Golgi signaling axis is a key mechanosensitive pathway that regulates Golgi organization and function, potentially linking matrix stiffness to tumor progression in breast cancer cells.

## INTRODUCTION

Breast cancer is the most common invasive cancer in women, affecting ∼12% of women worldwide (Shi et al., 2017). Physical cues, such as increased matrix stiffness, solid stress and interstitial fluid pressure drive breast tumor progression (Chai et al., 2023). Matrix stiffening is a key physical hallmark of breast cancer, which drives malignant transformation (Nia et al., 2020). Average breast tissue stiffness ranges from 0.5 kPa to 2.5 kPa, whereas breast tumor tissue stiffness could vary from 4 kPa to beyond 20 kPa (Liu et al., 2021).

Cells in their physiological niche can sense and respond to ECM mechanics, regulating migration, proliferation, differentiation and spreading (Martino et al., 2018). Mechanical cues are transmitted within cells, potentially influencing multiple pathways, including the dynamic organization of endomembrane organelles (Phuyal et al., 2020). Sensing of mechanical cues by cells is primarily done by integrins (Ffrench-Constant, 2004), which regulate cellular mechanotransduction pathways (Berrier and Yamada, 2007). Integrin-mediated cell–ECM adhesion governs key processes such as survival, growth and proliferation, and is frequently dysregulated in cancer (Schwartz, 1997). Previous work from our laboratory has shown that integrin-mediated cell–matrix adhesion is vital in regulating Golgi organization and functions in normal anchorage-dependent cells (Singh et al., 2018). Loss of matrix adhesion and integrin signaling causes marked Golgi disorganization in ‘normal’ anchorage-dependent mouse fibroblasts, driven by adhesion-dependent regulation of Arf1 activation (Singh et al., 2018).

Golgi organization and function are reported to be perturbed by established hallmarks of cancer (Petrosyan, 2015). Golgi morphology in certain cancer cell types is disorganized and/or dispersed (Makhoul et al., 2019). In some cancer cell types, the Golgi retains cisternal structure but loses its typical ribbon organization (Bajaj et al., 2022). The Golgi organization is ‘normal’ and compact in many cancer cells, as seen in non-transformed cells (Bajaj et al, 2022). Altered expression of Golgi-resident and structural proteins disrupts Golgi morphology and drives cancer progression, contributing to therapy resistance, epithelial–mesenchymal transition (EMT) and metastasis (Bui et al., 2021). In breast cancer, Golgi fragmentation is more prominent in triple-negative and invasive ductal carcinoma tissues than in hormone receptor-positive tumors or adjacent normal tissue, underscoring the pathological relevance of Golgi organization (Ghannoum et al., 2023). Notably, this remains the only study to date that examines this relationship in cells derived from individuals with breast tumors. However, whether Golgi disorganization actively drives tumorigenic traits or is merely a consequence of cell transformation remains unresolved. If ECM stiffness influences Golgi organization and function, it might shape cancer cell responses to mechanical cues, promoting malignancy. For example, on soft matrices, altered lipid and cholesterol synthesis affect Golgi rheology by inactivating lipin-1, which modulates diacylglycerol levels at the Golgi. (Romani et al., 2019). This reduces Arf1 recruitment to Golgi membranes, disrupting Golgi organization. Simultaneously, SCAP and SREBP proteins accumulate at the Golgi, activating SREBP1 and SREBP2 (also known as SREBF1 and SREBF2, respectively) and lipid synthesis (Romani et al., 2019).

In normal breast epithelial cells like MCF10A cells, the Golgi typically appears compact and perinuclear (Capaci et al., 2020). A study examining malignancy progression in MCF10A (non-tumorigenic), MCF10AT1 (pre-malignant) and MCF10CA1a (invasive) cells revealed that disruption of Golgi organization was corelated with increasing tumorigenic potential of these cells (Anandi et al., 2017). Consistent with this, MCF7 and MDA-MB-231 breast cancer cell lines differ markedly in hormone dependency, metabolism and epithelial-mesenchymal status, with previous studies reporting distinct Golgi organization between them (Kellokumpu et al., 2002). MDA-MB-231 cells have a predominant compact and organized Golgi, whereas MCF7 cells have a predominantly dispersed and disorganized Golgi (Kellokumpu et al., 2002).

As a first step to understanding the regulation of Golgi organization in these cells, independent studies have evaluated the differential expression of genes in the CCLE database that could regulate Golgi organization and/or function. This revealed differential expression of the receptor tyrosine kinase AXL (Colavito, 2020) in MDA-MB-231 (high) versus MCF7 (low) cells to be a putative regulator of differential Golgi organization in these cells (Joshi et al., 2025 preprint). In MDA-MB-231 cells, AXL localizes to the Golgi, essential for maintaining adhesion-dependent Golgi organization (Joshi et al., 2025 preprint). AXL is a known oncogene that enhances cancer cell proliferation, survival, migration and invasion, thus promoting overall cancer progression (Li et al., 2009). AXL overexpression and activation have also been reported in triple-negative breast cancers (Falcone et al., 2020). A study by Yang et al. also showed AXL to be involved in matrix-rigidity sensing by directly controlling the local mechanosensory contractions in focal adhesions without the involvement of its ligand Gas6 (Yang et al., 2016). Few studies have explored a possible crosstalk between AXL and the Golgi. A study has demonstrated that AXL localizes to the Golgi in highly migratory polarized triple-negative breast cancer cells (Hs578T and MDA-MB-231) and that its inhibition with R428 (Bemcentinib) impairs directed cell migration (Zajac et al., 2020). A genome-wide RNAi kinome screen in HeLa cells identified AXL as a potential regulator of Golgi organization (Chia et al., 2012). Only one study so far has reported a link between AXL and the small GTPase Arf1, suggesting that AXL might regulate Arf1 activation in breast cancer cells (Haines et al., 2015). However, this connection remains largely unexplored and needs further investigation. Together, these studies strongly suggest a possible role for AXL and Arf1 in regulating Golgi organization and function in breast cancer cells.

This study shows that changing matrix stiffness differentially affects Golgi organization in MDA-MB-231 versus MCF7 cells, regulated by their differential AXL expression. We further reveal stiffness-dependent regulation of AXL and Arf1 expression, which could support the idea that their regulatory interplay that drives stiffness-dependent Golgi organization. AXL and Arf1 control stiffness-dependent cell spreading, tubulin acetylation and Golgi-dependent cell surface glycosylation. Together, findings of the study identify the AXL-Arf1-Golgi axis as a novel mechanosensitive pathway that links matrix stiffness to Golgi organization and function, revealing a potential mechanism by which the tumor microenvironment could drive breast cancer progression.

## RESULTS

### Matrix stiffness-dependent regulation of Golgi organization, cell spreading and tubulin acetylation differs in MDA-MB-231 and MCF7 cells

Our previous study has established that cell–matrix adhesion is a crucial regulator of Golgi organization in ‘normal’ anchorage-dependent cells (Singh et al., 2018). In a recent related study, we also looked at the adhesion-dependent regulation of Golgi organization in anchorage-independent breast cancer cells, namely MDA-MB-231 and MCF7 cells using cis/medial and trans-Golgi markers (Joshi et al., 2025 preprint). In stable adherent MDA-MB-231 cells, the Golgi is organized but becomes disorganized upon loss of adhesion. However, in MCF7 cells, the Golgi is disorganized independently of its adhesion status (Joshi et al., 2025 preprint).

Knowing that matrix stiffening is a defining hallmark of breast tumor progression and a key regulator of adhesion signaling and cytoskeletal organization, we asked whether it could differentially influence Golgi organization in these two breast cancer cells. MDA-MB-231 and MCF7 cells, representing mesenchymal and epithelial-like states, respectively, also differ in their mechanical adaptability and invasiveness (Ouyang et al., 2025; Wullkopf et al., 2018). Therefore, comparing their Golgi organization response to matrix stiffness provides an opportunity to uncover how mechanoresponsive signaling affects Golgi organization and function in these two cell lines.

Prior studies have shown that Golgi organization in MDA-MB-231 affects cell–ECM adhesion and migration by modulating surface α5β1 integrin (Ahat et al., 2019), which might in turn influence anchorage-dependent signaling; however, despite these differences, both cell lines grow independently of anchorage and can support tumor formation (Mori et al., 2009). Studies have also reported that MDA-MB-231 and MCF7 cells differentially respond to adhesion and mechanosensory cues to regulate their migration (Vasudevan et al., 2020), drug uptake (Wang et al., 2017) and more.

**Fig. 1.**
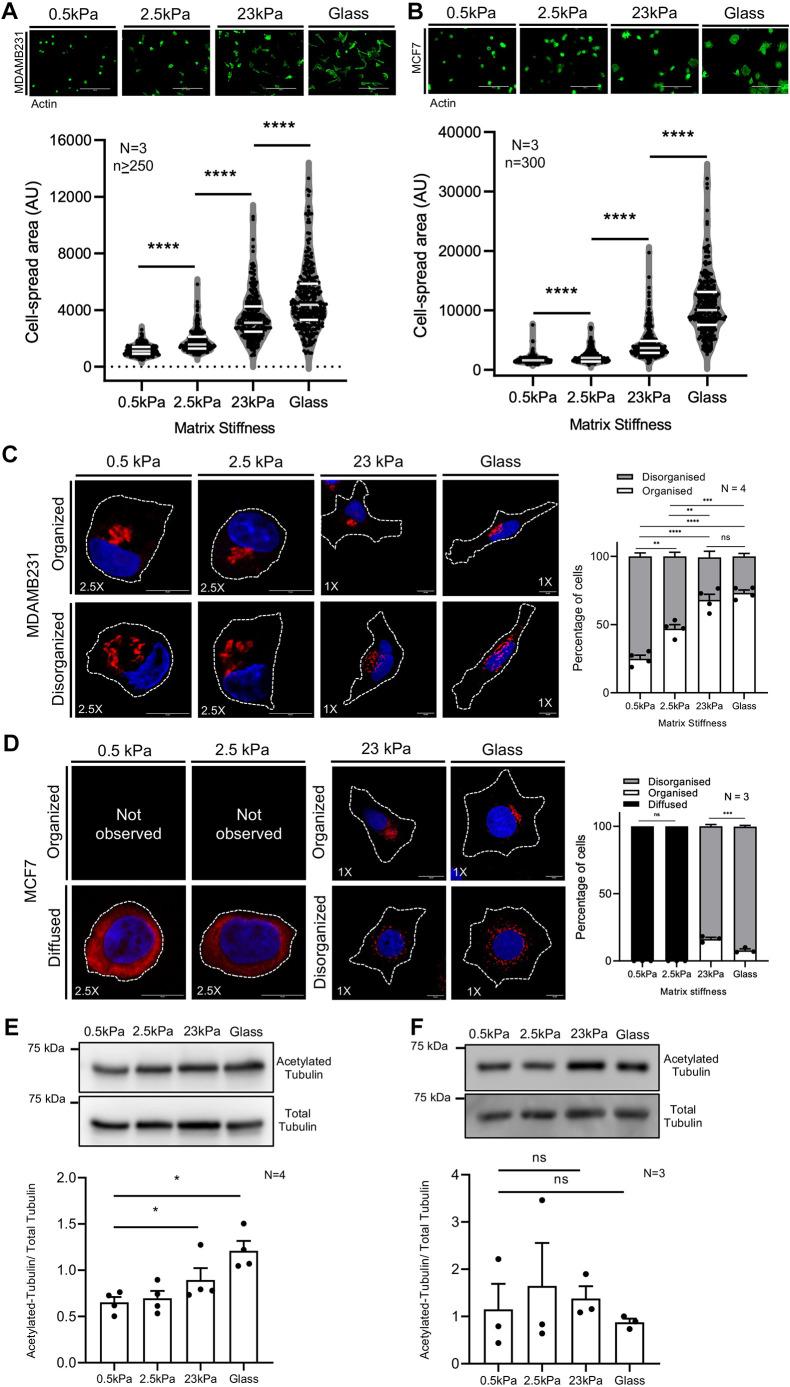
**Cell matrix adhesion-dependent matrix stiffness sensing differentially regulates Golgi organization, cell spreading and tubulin acetylation in MDA-MB-231 and MCF7 cells**. (A,B) Cross-section images of phalloidin stained MDA-MB-231 and MCF7 cells adherent on gels of various stiffnesses and glass. The graph highlights (white lines) the median and quartiles of cell spread area (arbitrary units, AU) for *n*≥250 cells, *N*=3 experiments. (C,D) Cross-section images of MDA-MB-231 and MCF7 cells immunostained for the cis-Golgi marker GM130 (red) and nucleus labeled with DAPI (blue) on gels of varying stiffness and glass. Percentage distribution profile of MDA-MB-231 and MCF7 cells with organized (white), disorganized (gray) and diffused with occasional puncta (black) Golgi, represented as mean±s.e.m. from *N*=4 (C) and *N*=3 (D) experiments. Cell outlines are marked with dotted lines. (E,F) Representative blots show acetylated tubulin and total tubulin levels in (E) MDA-MB-231 cells and (F) MCF7 cells on gels of varying stiffness and glass. Graph represents the ratio of densitometric band intensities as mean±s.e.m. from *N*=4 (E) and *N*=3 (F) experiments. The total tubulin blot shown in E is from the same experiment and hence also presented in Fig. S5A. *P≤0.05, **P≤0.01, ***P≤0.001, ****P≤0.0001; ns, not significant (one-way ANOVA multiple comparisons test with Tukey's method for error correction for distribution profiles and Mann–Whitney *U*-test for cell spread area and western blot analysis). Scale bars: 200 μm (A,B); 10 μm (C,D).

To investigate how matrix stiffness differentially influences their adhesion-dependent behavior, we first compared the adhesion-dependent spreading of MDA-MB-231 and MCF7 cells on collagen-coated (25 µg/ml collagen) 2D gels (0.5, 2.5 and 23 kPa) and glass to assess how matrix stiffness affects cell function. Both cell lines showed a stiffness-dependent increase in spread area (Fig. 1A,B), confirming their mechano-responsiveness. MCF7 cells spread more than MDA-MB-231, especially at higher stiffness (Fig. 1B), a trend further evident when their spread areas were compared together (Fig. S1A).

Correspondingly, the Golgi organization profile in MDA-MB-231 cells (detected using the cis-Golgi marker GM130; also known as GOLGA2) was also found to be stiffness dependent, predominantly disorganized on softer matrices (0.5 and 2.5 kPa) and increasingly organized on stiffer substrates (23 kPa and glass) (Fig. 1C). The profiling of cells as organized versus disorganized was corroborated by significant differences observed in their Golgi object numbers (Fig. S1B). These cells also exhibited a stiffness-dependent increase in Akt activation, a pathway known to act downstream of integrin-mediated phosphoinositide 3-kinase (PI3K) signaling (Fig. S1C). In MCF7 cells, the GM130-stained Golgi at lower stiffness (0.5 kPa and 2.5 kPa) was diffused throughout the cytosol with occasional puncta (Fig. 1D). On stiffer matrices (23 kPa and glass), the GM130-stained Golgi was predominantly disorganized, as confirmed by the distribution profile (Fig. 1D).

The Golgi is known to be a hub for non-centrosome microtubules to nucleate (Chabin-Brion et al., 2001), which could be affected when the Golgi organization is perturbed. In MDA-MB-231 cells, a stiffness-dependent increase in tubulin acetylation (and hence microtubule stability) (Wen et al., 2023) was observed (Fig. 1E) that could reflect the impact of their Golgi organization being restored at higher stiffness. Notably, unlike MDA-MB-231 cells, MCF7 cells do not exhibit a stiffness-dependent increase in tubulin acetylation (Fig. 1F), possibly implicating the lack of stiffness-dependent Golgi organization in these cells. In MCF7 (and MDA-MB-231 cells), mannosidase-II tagged with GFP (ManII–GFP, a cis and medial Golgi marker) and GalTase-RFP (a trans-Golgi marker) transfection efficiency was low, making it challenging to have enough transfected cells on 2D PA gels to evaluate the Golgi phenotype, particularly on softer matrices. Interestingly, MCF7 and MDA-MB-231 cells, with primarily disorganized and organized Golgi phenotypes, respectively, also contain a secondary population showing the opposite phenotype. The cause and impact of this heterogeneity on cellular mechano-responsiveness remain unclear. Identifying what mediates matrix stiffness-dependent Golgi regulation is a crucial first step.

### Differentially expressed AXL regulates stiffness-dependent Golgi organization and cell spreading in MDA-MB-231 cells

AXL, a receptor tyrosine kinase known to be involved in cellular rigidity sensing (Yang et al., 2016), is differentially expressed in MDA-MB-231 (high expression) and MCF7 cells (no expression) (Fig. S2A). In MCF10A non-tumorigenic breast epithelial cells, AXL expression is lower than in MDA-MB-231 cells but distinctly higher than MCF7 (Fig. S2A). Along with its plasma membrane localization, AXL is reported to localize at the Golgi in migrating Hs578t and MDA-MB-231 cells (Zajac et al., 2020), which we also observed in MCF10A cells (Fig. S2B). Treatment of MCF10A cells with the selective AXL inhibitor R428 (Holland et al., 2010) caused significant Golgi disorganization, as confirmed by the percentage distribution profile (Fig. S2C). This treatment also led to a modest but significant reduction in tubulin acetylation (Fig. S2D).

Motivated by these observations, we examined AXL localization relative to the cis-Golgi marker GM130 in MDA-MB-231 cells on matrices of increasing stiffness – 2.5 kPa (soft), 23 kPa, and glass (high stiffness). As stiffness increased, the Golgi (GM130) became more organized (at 23 kPa and glass), showing enhanced colocalization with AXL, which is confirmed by Pearson's correlation coefficient analysis (Fig. S2E). In 2.5 kPa gels with disorganized Golgi, AXL overlap with GM130 was significantly less (Fig. S2E). These results show stiffness-dependent AXL localization to the organized Golgi, suggesting a potential role for AXL at the Golgi that we further explored in MDA-MB-231 cells.

**Fig. 2.**
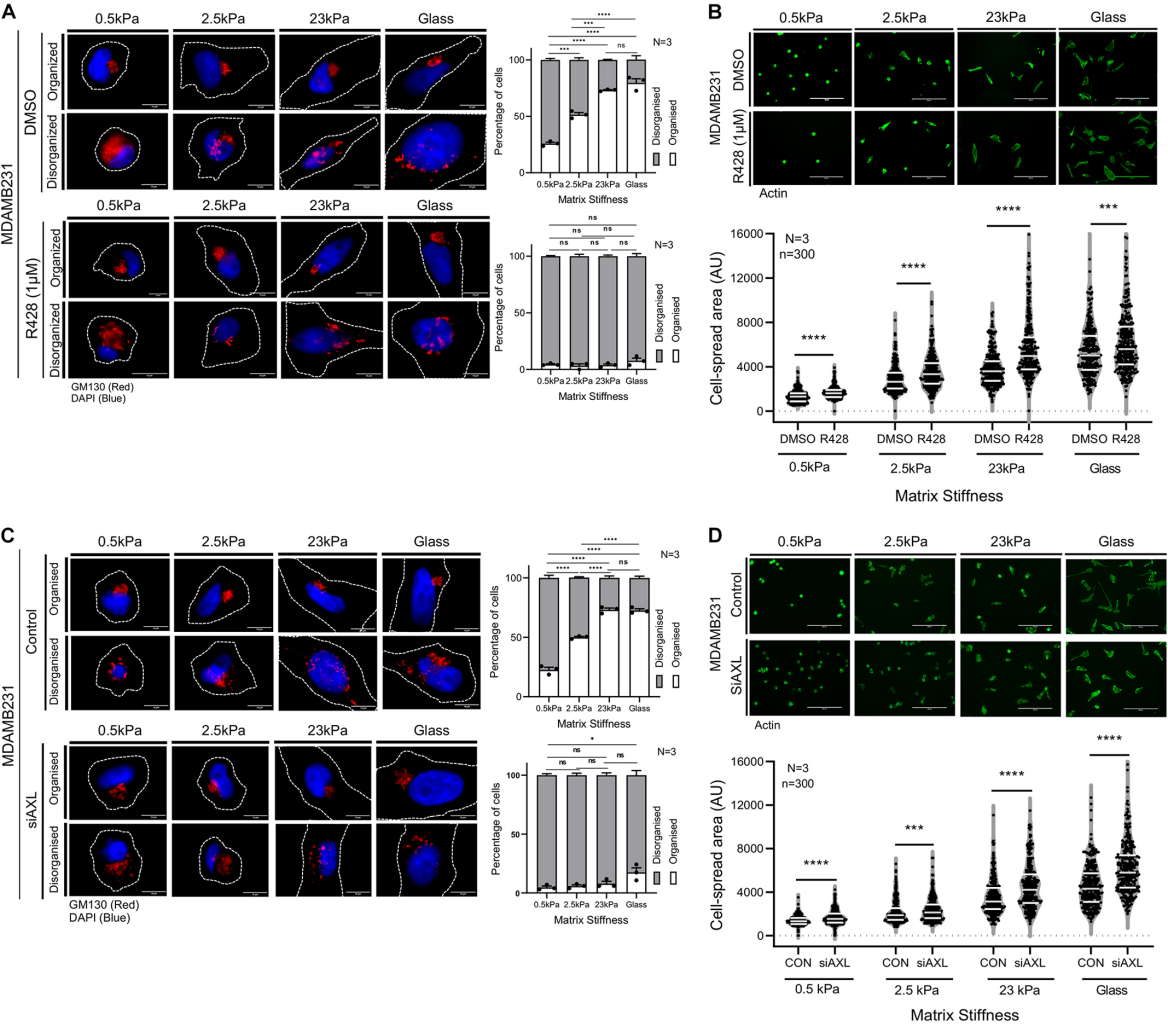
**AXL activation and expression regulate stiffness-dependent Golgi organization and cell spreading in MDA-MB-231 cells.** (A) Cross section images of DMSO and R428 treated MDA-MB-231 cells immunostained for the cis-Golgi marker GM130 (red) and nucleus labeled with DAPI (blue) on gels of various stiffnesses and glass. Graph represents percentage distribution profile of cells with organized and disorganized Golgi as mean±s.e.m. from *N*=3 experiments. (B) Cross-section images of phalloidin stained DMSO and R428 treated MDA-MB-231 cells on gels of varying stiffness and glass. The graph highlights (white lines) the median and quartiles of cell area for *n*=300 cells from *N*=3 experiments. (C) Cross section images of control and AXL knockdown (siAXL) MDA-MB-231 cells immunostained for the cis Golgi marker-GM130 (red) and nucleus labelled with DAPI (blue) on gels of varying stiffness and glass. Graph represents percentage distribution of cells with organized and disorganized Golgi shown as mean±s.e.m. from *N*=3 experiments. (D) Cross-section images of phalloidin stained control and AXL knockdown (siAXL) MDA-MB-231 cells on gels of various stiffnesses and glass. The graph highlights (white lines) the median and quartiles of cell area for *n*=300 cells from *N*=3 experiments. *P≤0.05, ***P≤0.001, ****P≤0.0001, ns, not significant (one-way ANOVA multiple comparisons test with Tukey's method for the distribution profiles and Mann–Whitney *U*-test for cell spread area analysis). Cell outlines are marked with dotted lines in A and C. AU, arbitrary units. Scale bars: 10 μm (A,C); 200 μm (B,D).

Targeting AXL with R428 in MDA-MB-231 cells disrupted stiffness-dependent Golgi organization in these cells (Fig. 2A). The Golgi was predominantly disorganized across all stiffnesses in inhibitor-treated cells (Fig. 2A). R428-mediated disruption of stiffness-dependent Golgi organization in MDA-MB-231 was accompanied by increased stiffness-dependent cell spreading at all stiffnesses (Fig. 2B), which was further evident when plotted together as a trend line (Fig. S2F). The increase in spreading of R428-treated cells is prominent at higher stiffnesses (23 kPa and glass) (Fig. S2F). R428 treatment of MDA-MB-231 cells at 23 kPa and on glass caused a reduction in Akt activation downstream of AXL that was regulated by matrix stiffness, confirming its effect (Okimoto and Bivona, 2015) (Fig. S2G). To determine whether the effect of R428 on Golgi organization was mediated through Akt inhibition, we tested the impact of a selective Akt inhibitor (Akt inhibitor VIII) on MDA-MB-231 cells. This inhibitor treatment effectively suppressed Akt activation (Fig. S2H) without altering total AXL levels (Fig. S2I). However, direct Akt inhibition did not cause Golgi disorganization in these cells (Fig. S2J). These results suggest that the Golgi disorganization observed with R428 treatment is not a consequence of its effect on Akt activation.

Next, we tested whether siRNA-mediated AXL knockdown, like R428 treatment, also affects Golgi organization and cell spreading. Using a validated siRNA (Li et al., 2009; Holland et al., 2005), we confirmed AXL knockdown in MDA-MB-231 cells (Fig. S2K). Like R428 treatment, AXL knockdown disrupted stiffness-dependent Golgi organization across all stiffness (Fig. 2C) and caused increased cell spreading (Fig. 2D), which was evident when plotted together as a trend line (Fig. S2L). This increase in spreading is prominent at higher stiffnesses (23 kPa and glass) (Fig. S2G).

Together, these studies suggest that both expression and activation of AXL contribute to stiffness-dependent changes in Golgi organization in MDA-MB-231 cells. Given that Golgi-mediated trafficking and processing have been previously associated with cell adhesion (Ahat et al., 2019), it is plausible that these alterations in Golgi organization downstream of AXL could influence the changes observed in adhesion-dependent cell spreading. In response to changing matrix stiffness, this regulation could reflect in subtle changes of cell functions as well.

### AXL expression regulates Golgi organization in MCF7 cells

**Fig. 3.**
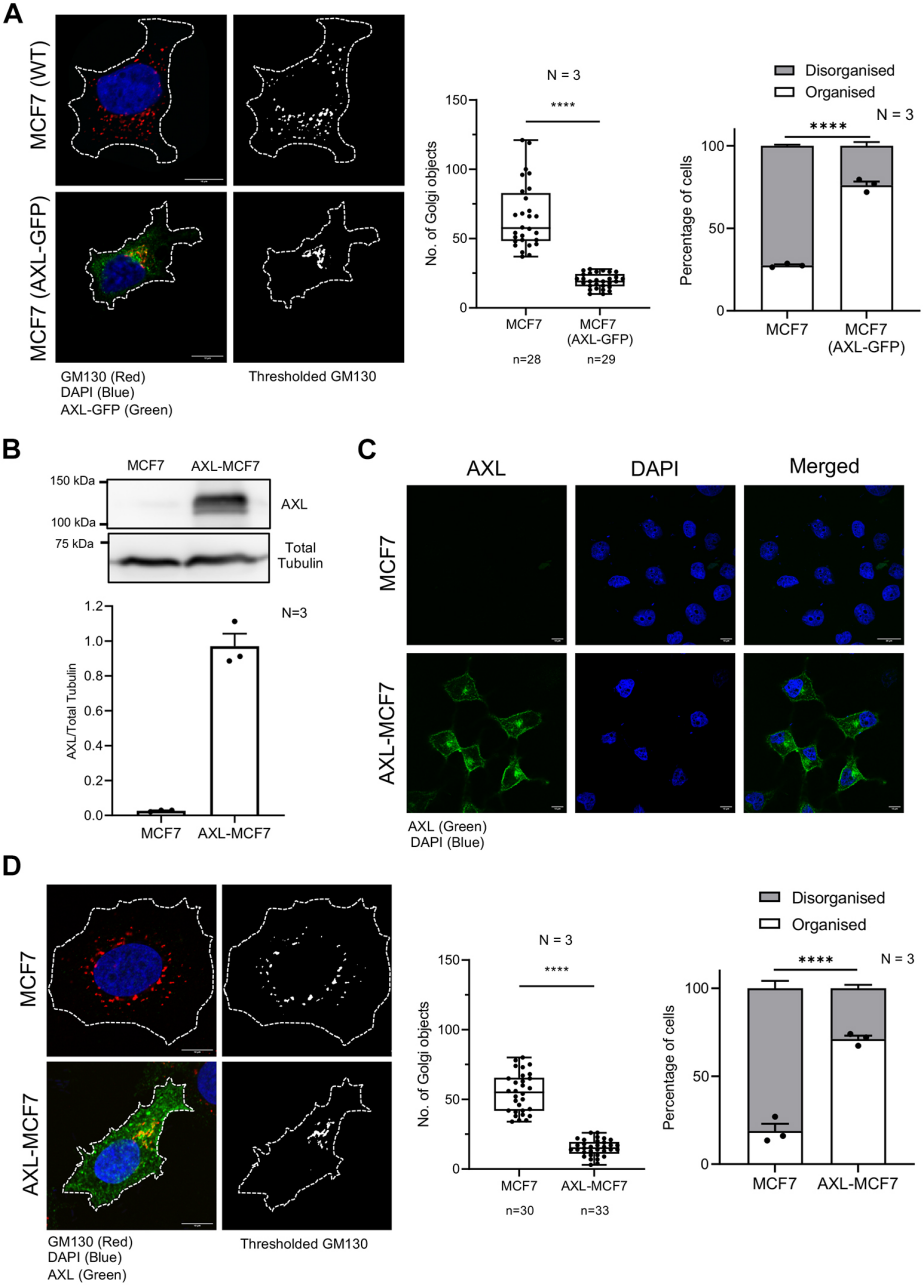
**AXL expression regulates Golgi organization in adherent MCF7 cells.** (A) Cross-section images for wild-type (WT) MCF7 and MCF7 (AXL–GFP) cells on glass, immunostained for the GM130 marker (red) and nucleus labeled with DAPI (blue) showing predominant Golgi organization phenotype. The black-and-white images show thresholded GM130 Golgi objects. In box-and-whisker plots for object counts, the box represents the 25–75th percentiles, and the median is indicated. The whiskers show minimum and maximum for cis-Golgi object counts per cell (*n*≥25). The bar graph shows the percentage distribution profile for cells with organized and disorganized Golgi representing mean±s.e.m. from *N*=3 experiments. (B) Representative blots for AXL and total tubulin in MCF7 and AXL-MCF7 cells. Graph represents the ratio of densitometric band intensities as mean±s.e.m. from *N*=3 experiments. (C) MCF7 and AXL-MCF7 cells immunostained for AXL (green) and the nucleus labeled with DAPI (blue). (D) Cross-section images of MCF7 and AXL-MCF7 cells on glass, immunostained for AXL (green), the cis-Golgi marker GM130 (red) and nucleus labeled with DAPI (blue) showing predominant Golgi organization phenotype. The black-and-white images show thresholded GM130-Golgi objects. In box-and-whisker plots for object counts, the box represents the 25–75th percentiles, and the median is indicated. The whiskers show minimum and maximum for cis-Golgi object counts per cell (*n*=30). The bar graph shows the percentage distribution profile for cells with organized and disorganized Golgi representing mean±s.e.m. from *N*=3 experiments. ****P≤0.0001 (one-way ANOVA multiple comparisons test with Tukey's method for the distribution profiles and Mann–Whitney *U*-test and object count analysis). Cell outlines are marked with dotted lines in A and D. Scale bars: 10 μm.

In MCF7 cells lacking AXL expression, the pronounced disorganization of the Golgi provides a suitable context to investigate the mechanosensitive regulation of Golgi organization by AXL. Transient pEGFP-N1-AXL expression in MCF7 cells reorganized the Golgi, as seen in the percentage distribution profile, and significantly reduced GM130-stained Golgi object numbers compared to untransfected cells (Fig. 3A). This is reflected in a significant change in the percentage distribution profile of the ‘organized’ Golgi phenotype in transfected versus un-transfected cells (Fig. 3A). In AXL-expressing MCF7 (AXL-MCF7) cells, the Golgi was not radially distributed around the nucleus, as in MCF7 cells, but instead showed a more compact perinuclear localization with a marked reduction in Golgi object numbers (Fig. 3A).

Furthermore, AXL was stably reconstituted in MCF7 cells via retroviral transduction. Notably, the overall morphology of AXL-expressing MCF7 cells remained broadly comparable to that of parental MCF7 cells (Fig. S3A). AXL expression and localization in these cells were validated using western blotting and immunostaining (Fig. 3B,C). Stable AXL expression in MCF7 cells (AXL-MCF7) also restored Golgi organization accompanied by a significant reduction in Golgi object numbers relative to MCF7 cells (Fig. 3D). This is reflected in a marked shift in the percentage of cells with an ‘organized’ Golgi phenotype in stable AXL-expressing MCF7 (AXL-MCF7) cells compared to what is seen for parental MCF7 cells (Fig. 3D). Golgi organization in stable AXL-MCF7 cells was comparable to transient AXL expression (Fig. 3A,D). This was further confirmed using ManII–GFP (cis/medial) and GalTase–RFP (trans) Golgi markers (Fig. S3B). The distinctly re-organized Golgi in AXL-expressing MCF7 cells was less compact than that of MDA-MB-231 cells, which could reflect differences in relative AXL expression. This was further reflected by the AXL localization, which showed only a partial overlap with both cis-Golgi (GM130) and trans-Golgi (GalTase-RFP) compartments (Fig. S3C) in those cells. These results highlight AXL expression as necessary for the differential Golgi organization in breast cancer cells.

**Fig. 4.**
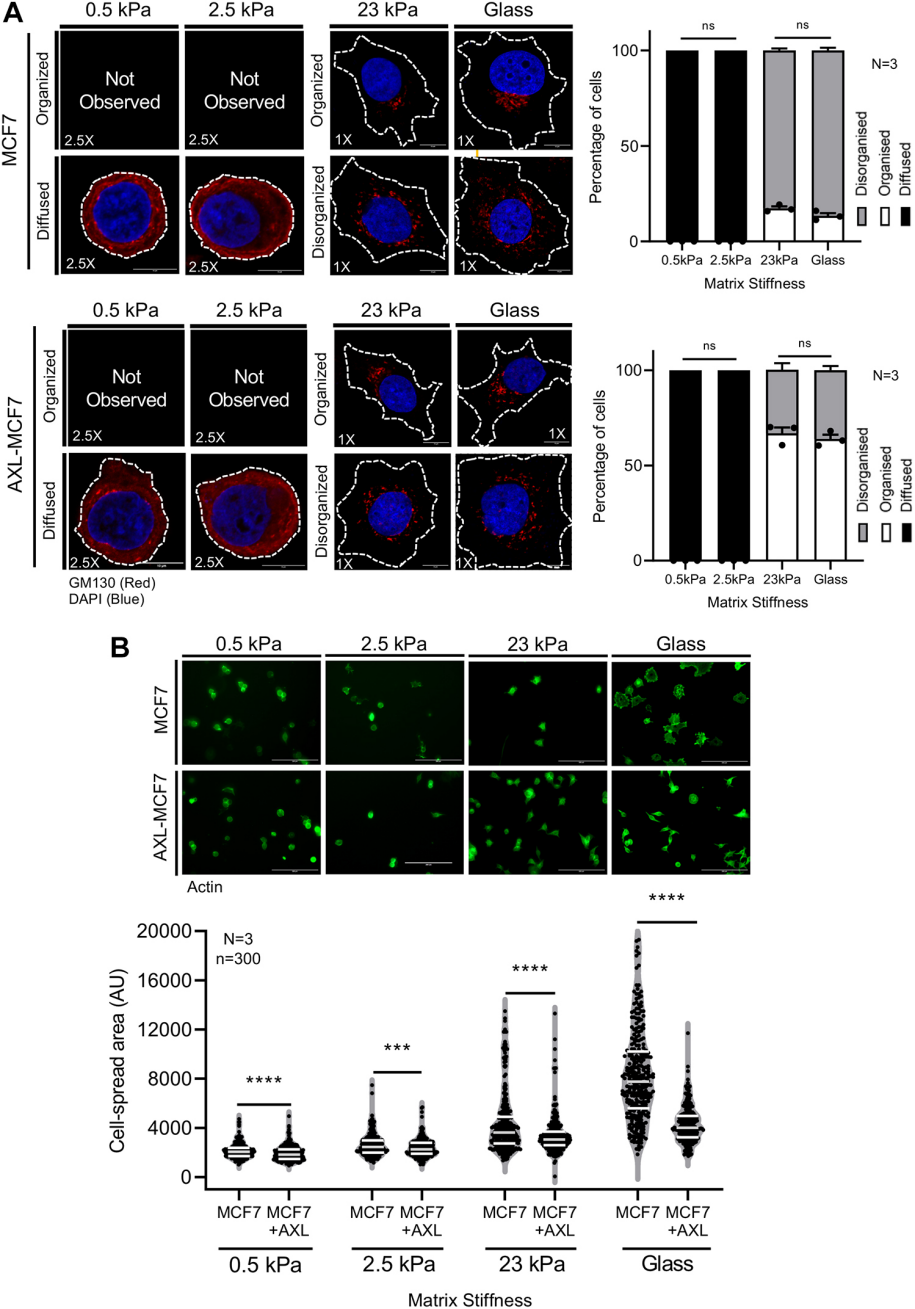
**AXL expression regulates Golgi organization and cell spreading in MCF7 cells on stiffer matrices.** (A) Cross section images of MCF7 and AXL-MCF7 cells immunostained for the GM130 marker (red) and nucleus labeled with DAPI (blue) on gels of various stiffnesses and glass. Graph represents percentage distribution profile of cells with organized and disorganized Golgi as mean±s.e.m. from *N*=3 experiments. Cell outlines are marked with dotted lines. (B) Cross-section images of phalloidin stained MCF7 and AXL-MCF7 cells on gels of various stiffnesses and glass. The graph highlights (white lines) the median and quartiles for *n*=300 cells from *N*=3 experiments. ***P≤0.001, ****P≤0.0001; ns, not significant (one-way ANOVA multiple comparisons test with Tukey's method for the distribution profiles and Mann–Whitney *U*-test for cell spread area analysis.). Scale bars: 10 μm (A); 200 μm (B).

Next, we examined whether AXL-MCF7 cells respond to changes in matrix stiffness similarly to MDA-MB-231 cells. In parental MCF7 cells, the GM130-stained Golgi at lower stiffness (0.5 kPa and 2.5 kPa) appeared diffusely distributed throughout the cytosol, with occasional puncta (Fig. 1E). On stiffer matrices (23 kPa and glass), the Golgi phenotype was predominantly disorganized (Fig. 1E, Fig. 4A). In AXL-MCF7 cells, the Golgi phenotype was restored to being predominantly ‘organized’ at 23 kPa and on glass (Fig. 4A). At lower stiffnesses (0.5 and 2.5 kPa), the Golgi organization stayed diffused with occasional puncta (Fig. 4A). This might reflect either the influence of AXL expression levels in AXL-MCF7 cells or that AXL is not the primary regulator of Golgi organization at lower stiffness. We next examined whether AXL expression affects stiffness-dependent cell spreading in MCF7 cells. AXL-MCF7 cells showed significantly reduced spreading across all stiffnesses (Fig. 4B; Fig. S4A). These findings suggest that the influence of AXL on stiffness-dependent spreading in MCF7 cells is largely Golgi-independent at lower stiffnesses.

Together, the above results suggest AXL expression and/or activation regulates stiffness-dependent cell spreading in MCF7 (Fig. 4B) and MDA-MB-231 cells (Fig. S2C,E), possibly regulated by adhesion-dependent signaling. Adhesion-dependent regulation of Arf1 is known to control Golgi organization (Singh et al., 2018). Arf1 activation is shown to be regulated by actomyosin contractility in cells (Romani et al., 2019). Inhibition of ROCK and MLCK proteins in cells is known to cause a drop in Arf1 activation (Romani et al., 2019). This could mimic their behavior on a softer matrix. Arf1 has also been reported to interact with AXL, and its inhibition by R428 in MDA-MB-231 cells increases Arf1 activation (Haines et al., 2015). This potential crosstalk between AXL and Arf1 presented an intriguing axis for further investigation.

### Role of AXL-Arf1 crosstalk in stiffness-dependent regulation of Golgi organization and functions

**Fig. 5.**
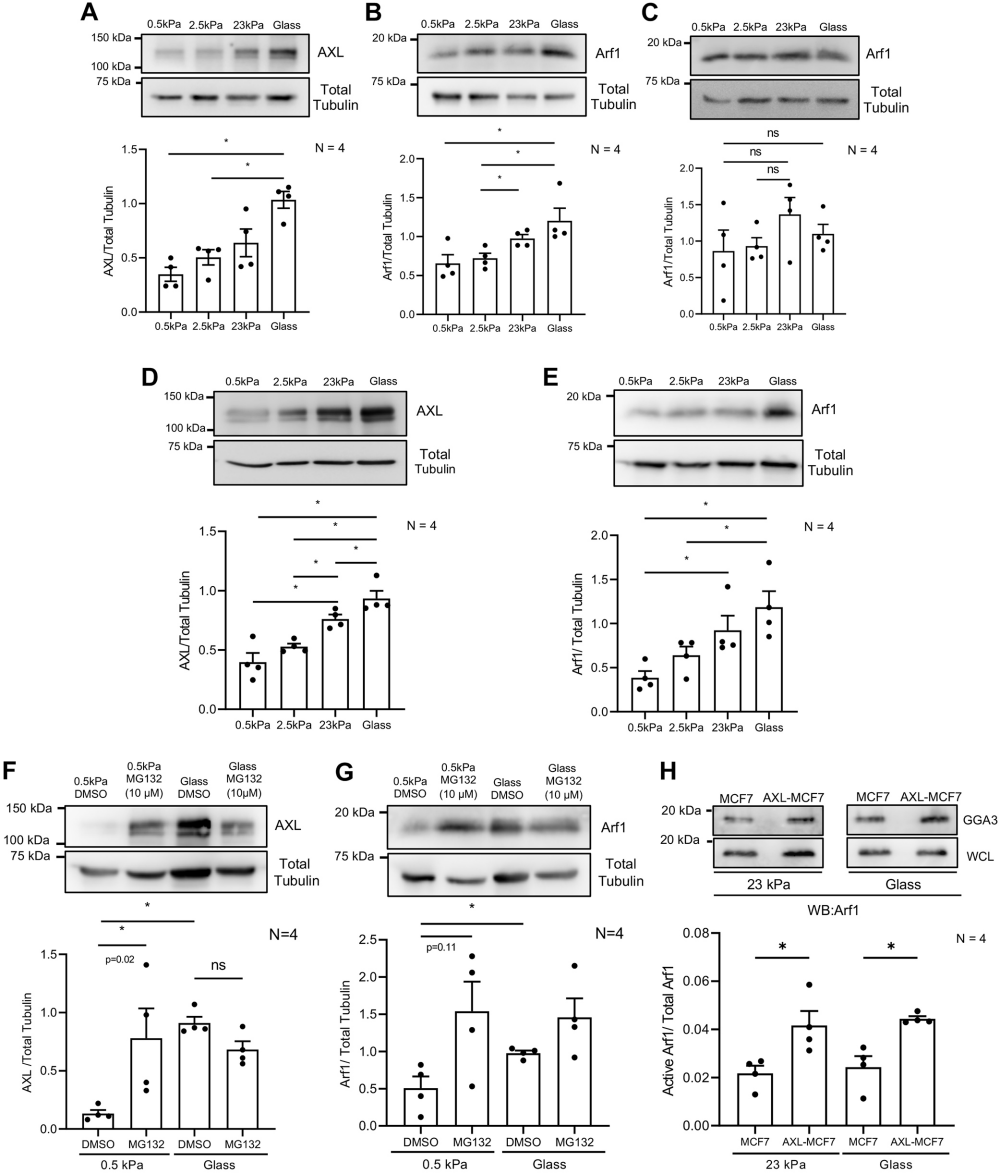
**Stiffness dependent expression of AXL and Arf1 regulates stiffness-dependent Golgi organization in breast cancer cells.** (A–E) Representative blots for AXL and total tubulin in (A) MDA-MB-231, (D) AXL-MCF7 cells and Arf1 and total tubulin in (B) MDA-MB-231, (C) MCF7 and (E) AXL-MCF7 cells on gels of varying stiffness and glass. (F,G) Representative blots for (F) AXL and (G) Arf1 and total tubulin in DMSO and MG132 treated AXL-MCF7 cells on 0.5 kPa gel and glass. Graphs in A–G represent the ratio of densitometric band intensities (AXL/total tubulin and Arf1/total tubulin) as mean±s.e.m. from *N*=4 experiments. (H) Active Arf1 pulled down using GST–GGA3 (GGA3) and total Arf1 in whole cell lysate (WCL) from MCF7 and AXL-MCF7 cells on 23 kPa gel and glass. Graph represents the ratio of densitometric band intensities as mean±s.e.m. from *N*=4 experiments. *P≤0.05; ns, not significant or *P*-values when indicated (Mann–Whitney *U*-test).

Previous studies have shown Arf1 activation and Golgi localization are vital for its organization and function (Donaldson et al., 2005; Altan-Bonnet et al., 2003; Jackson and Bouvet, 2014). Laboratory studies have shown that adhesion-dependent Arf1 activation regulates Golgi organization and function in anchorage-dependent mouse fibroblasts (Singh et al., 2018). Co-immunoprecipitation studies in MDA-MB-231 cells have further revealed that Arf1 physically associates with AXL, supporting the idea that there is a direct interaction between the two proteins (Haines et al., 2015). This highlights the adhesion-AXL-Arf1-Golgi pathway as a particularly intriguing regulatory axis. To investigate, we first assessed AXL and Arf1 expression in MDA-MB-231 cells across various matrix stiffnesses, finding that AXL levels increased in a stiffness-dependent manner (Fig. 5A). This was reflected in a stiffness-dependent increase in net phosphorylated (p)AXL (Y702) levels (relative to tubulin), which is lost upon normalization to total AXL (Fig. S5A). Given the variable reports on the impact of Y702 phosphorylation on AXL activation, its interpretation here remains uncertain (Holland et al., 2010; Chen et al., 2018; Wu et al., 2021). Independent studies from the laboratory indicate that the AXL inhibitor R428 increases pAXL (Y702) levels in MDA-MB-231 and A549 cells (Joshi et al., 2025 preprint). Total Arf1 levels also increased in a stiffness-dependent manner in MDA-MB-231 cells (Fig. 5B), but this effect was absent in MCF7 cells lacking AXL (Fig. 5C). Such stiffness-dependent regulation of both AXL and Arf1 further supports the possibility of their crosstalk regulating Golgi organization.

To test this crosstalk, we evaluated the matrix stiffness-dependent regulation of AXL-MCF7 cells. AXL-MCF7 cells showed a matrix stiffness-dependent increase in AXL (Fig. 5D) and Arf1 levels (Fig. 5E), like MDA-MB-231 cells (Fig. 5A,B). The stiffness-dependent increase in AXL levels in stable-expressing cells, unlike endogenous AXL in MDA-MB-231, suggests post-transcriptional or translational regulation, possibly through mRNA stability (Cho et al., 2019; Cho et al., 2016) or protein degradation (Krishnamoorthy et al., 2013).

To test the role of proteasomal degradation in this regulation, AXL-MCF7 cells on 0.5 kPa gels (which had the lowest AXL levels) were treated with MG132 (10 µM), which restored AXL levels observed to the values observed on glass (Fig. 5F) accompanied by an increase in overall polyubiquitination (Fig. S5B). No significant change occurred for cells on glass with MG132 versus DMSO (Fig. 5F). This supports proteasomal degradation being the leading cause of low AXL on 0.5 kPa. MG132 also caused an increase in Arf1 levels (Fig. 5G) compared to DMSO, suggesting that proteasomal degradation and/or elevated AXL might influence Arf1 levels in these cells.

The correlated expression of AXL and Arf1 might support Golgi organization at higher stiffness (23 kPa and glass). As Arf1 activation is key for adhesion-dependent Golgi organization (Singh et al., 2018), the stiffness-driven rise in total Arf1 likely increases active Arf1 levels. AXL might also influence Arf1 activation. Using a GST–GGA3 pulldown assay, we found active Arf1 levels were significantly higher in AXL-MCF7 than MCF7 cells on 23 kPa and glass (Fig. 5H). In these cell lysates, active Arf1 pulled down using GST–GGA3 also co-precipitated AXL, indicating a potential association between AXL and active Arf1 (Fig. S5C). Consistent with our findings, Haines et al. (2015) reported an interaction between AXL and Arf1, demonstrated through immunoprecipitation of total Arf1 (Haines et al., 2015).

**Fig. 6.**
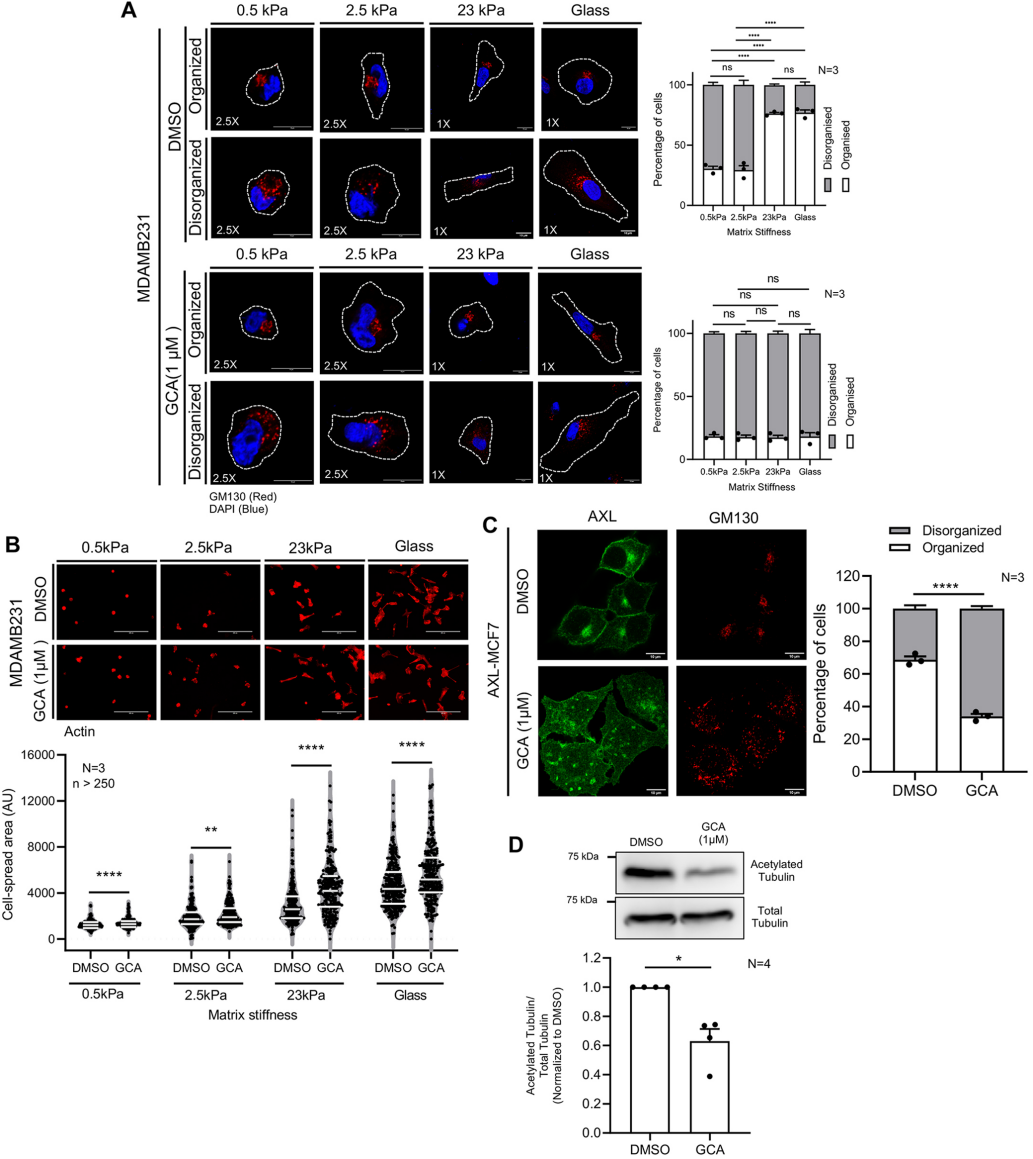
**Arf1 activation regulates matrix stiffness-dependent Golgi organization and cell spreading in breast cancer cells.** (A) Cross section images of DMSO and Golgicide A (GCA)-treated MDA-MB-231 cells immunostained for the GM130 marker (red) and the nucleus labeled with DAPI (blue) on gels of various stiffnesses and glass. Graph represents percentage distribution profile of cells with organized and disorganized Golgi as mean±s.e.m. from *N*=3 experiments. Cell outlines are marked with dotted lines. These representative control images were used for Golgi object count quantification, of cells with organized or disorganized Golgi phenotypes in Fig. S1B. (B) Cross-section images of phalloidin stained DMSO and GCA treated MDA-MB-231 cells on gels of various stiffnesses and glass. The graph highlights (white lines) the median and quartiles of cell area for *n*≥250 cells from *N*=3 experiments. (C) Cross-section images for predominant Golgi organization phenotype for DMSO- and GCA-treated AXL-MCF7 cells immunostained for GM130 (red) and AXL (green) on glass. Graphs represent percentage distribution profile of cells with organized and disorganized Golgi as mean±s.e.m. from *N*=3 experiments. (D) Representative blots for acetylated tubulin and total tubulin levels in DMSO- and GCA-treated AXL-MCF7 cells. Graph represents the ratio of densitometric band intensities normalized to DMSO control as mean±s.e.m. from *N*=4 experiments. *P≤0.05; **P≤0.01; ****P≤0.0001; ns, not significant (one-way ANOVA multiple comparisons test with Tukey's method for the distribution profiles, Mann–Whitney *U*-test for cell area analysis and single sample Wilcoxon *t*-test for western blots analysis normalized with respect to control). Scale bars: 10 μm (A,C); 200 μm (B).

To further dissect the role of Arf1 in AXL-Arf1-dependent mechanosensitive regulation of Golgi organization, we treated MDA-MB-231 cells with 1 µM Golgicide A (GCA), an inhibitor of the Arf1 GEF GBF1 (Sáenz et al., 2009), on matrices of varying stiffness. This inhibition disrupted Golgi organization (Fig. 6A) and enhanced cell spreading (Fig. 6B; Fig. S6A) across all stiffnesses, closely mirroring the effects of AXL inhibition by R428 (Fig. 2B,D).

As AXL expression in AXL-MCF7 cells regulates Arf1 activation (Fig. 5H), we tested how GCA-mediated Arf1 inhibition affects the Golgi in these cells. Arf1 and its GEF GBF1 were expressed at similar levels in MCF7 and AXL-MCF7 cells (Fig. S6B,C). GCA-mediated Arf1 inhibition caused Golgi disorganization in AXL-MCF7 cells (Fig. 6C) accompanied by a significant drop in tubulin acetylation (Fig. 6D). This reiterates the possible functional link between Golgi organization and tubulin acetylation in these cells. Qualitative analysis showed that active Arf1 (ABD–GFP) (Kumari and Mayor, 2008) and GBF1 (Meissner et al., 2018) colocalized more with GM130-stained Golgi in AXL-MCF7 cells on glass than in the disorganized Golgi of MCF7 cells (Fig. S6D,E). These findings point to an AXL-GBF1-Arf1 crosstalk at the Golgi, with AXL-dependent GBF1 and active Arf1 recruitment warranting further studies.

**Fig. 7.**
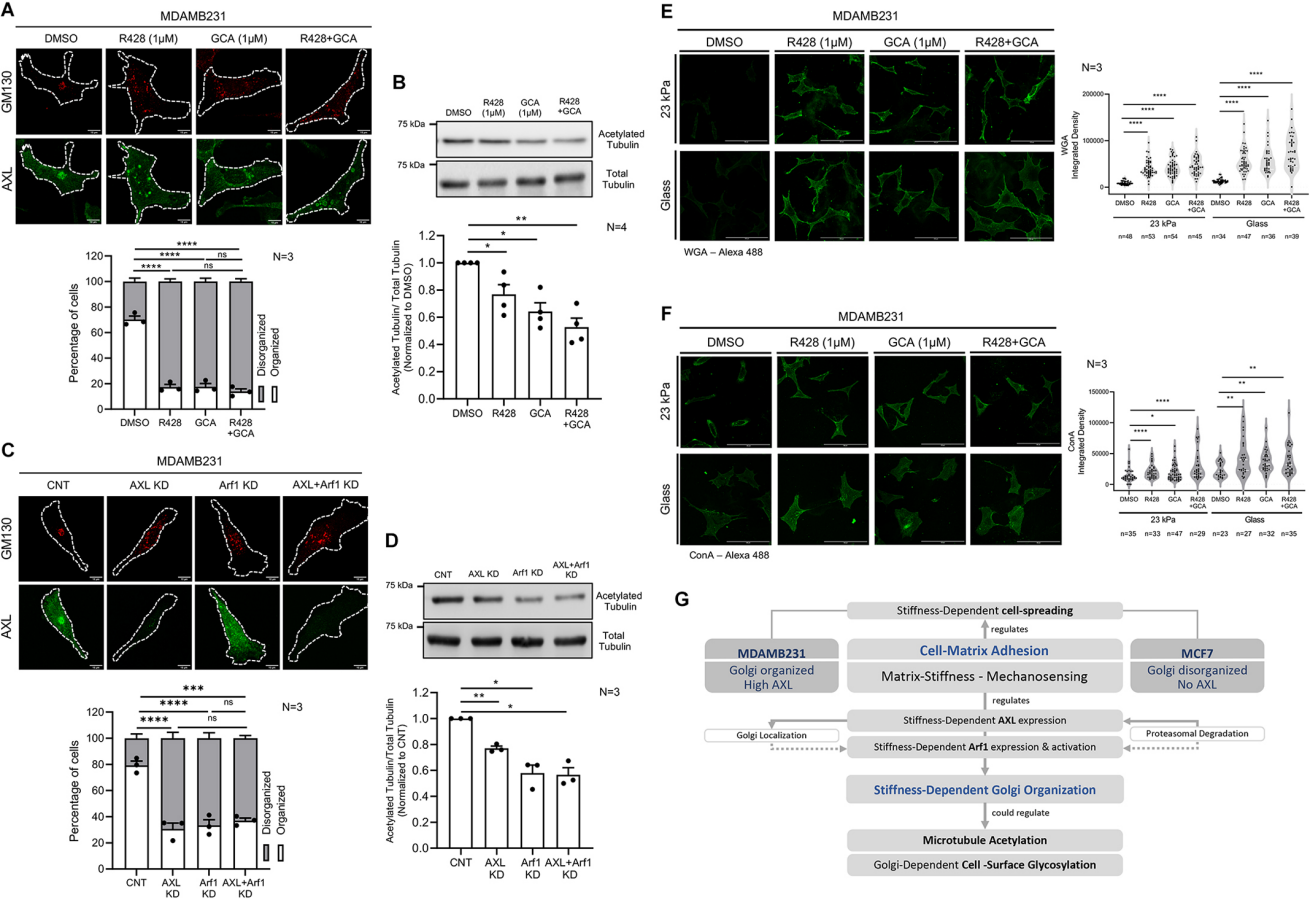
**AXL- and Arf1-mediated regulation of Golgi organization and function, tubulin acetylation and cell surface glycosylation in MDA-MB-231 cells.** (A,C) Cross-section images for predominant Golgi organization phenotype for (A) DMSO, R428, GCA and R428+GCA treated and (C) control, AXL knockdown (KD), Arf1 knockdown (KD), and AXL+Arf1 KD MDA-MB-231 cells immunostained for GM130 (red) and AXL (green) on glass. Graph represents percentage distribution profile for cells with organized and disorganized Golgi as mean±s.e.m. from *N*=3 experiments. (B,D) Representative blots for acetylated tubulin and total tubulin in (B) DMSO, R428, GCA and R428+GCA and (D) control, AXL KD, Arf1 KD, and AXL+Arf1 KD MDA-MB-231 cells on glass. Graph represents the ratio of densitometric band intensities normalized to control as mean±s.e.m. from (B) *N*=4 and (D) *N*=3 experiments. (E,F) Cross-section images for DMSO, R428, GCA and R428+GCA treated MDA-MB-231 cells stained with (E) WGA–Alexa-Fluor-488 and (F) ConA–Alexa-Fluor-488 on 23 kPa gel and glass. Graphs represent integrated density per cell plotted as mean±s.e.m. for multiple cells (*n*) in each condition, from *N*=3 experiments. *P≤0.05; **P≤0.01; ***P≤0.001; ****P≤0.0001; ns, not significant (single sample Wilcoxon *t*-test for western blots analysis normalized with respect to control and Mann–Whitney *U*-test for WGA and ConA lectin intensity profiles). Cell outlines are marked with dotted lines in A and C. Scale bars: 10 μm (A,C); 100 μm (E,F). (G) Schematic shows a model of how differential AXL expression in MDA-MB-231 and MCF7 cells regulates stiffness-dependent Golgi organization and function via the AXL-Arf1 axis, impacting microtubule acetylation and Golgi-mediated cell surface glycosylation.

To determine whether AXL and Arf1 act via a common pathway, we compared their individual and combined inhibition (R428 and GCA) and siRNA knockdown effects on Golgi organization in MDA-MB-231 cells on glass. Targeting either Arf1 or AXL (GCA+R428 or siArf1+siAXL; Fig. S7C), caused similar Golgi disorganization (Fig. 7A,C), and dual targeting showed no additive effect, suggesting they regulate Golgi organization through a shared pathway. When these cells were evaluated for their acetylated tubulin levels, targeting Arf1 caused a slightly greater reduction in acetylated tubulin levels than targeting AXL alone (Fig. 7B,D). Combined targeting produced effects that were similar to those seen upon Arf1 inhibition or knockdown. This suggests that, alongside Golgi disorganization, Arf1 may also regulate tubulin acetylation via a Golgi-independent mechanism, as previously reported (Zhang et al., 2024).

As a direct measure of Golgi function, we assessed the impact of inhibitor treatments on the cell surface glycosylation of MDA-MB-231 cells on 23 kPa and glass. Using fluorescently tagged wheatgerm agglutinin (WGA; for sialic acid and GlcNAc) and concanavalin A (ConA for mannose-rich N-glycans), we found that inhibiting AXL (R428), Arf1 (GCA) or both increased lectin binding, correlating with Golgi disorganization (Fig. 7A,C,E,F). This confirms that AXL- and Arf1-dependent Golgi disorganization alters Golgi-mediated cell surface glycosylation, potentially impacting cell receptor functions.

Our findings hence reveal that matrix stiffness-mediated mechanosensing drives Golgi organization through an AXL-Arf1 axis, linking cell–matrix adhesion to tubulin acetylation and Golgi-dependent cell surface glycosylation in breast cancer cells (Fig. 7G).

## DISCUSSION

Among the several well-established hallmarks of breast cancers, stiffening of the tumor matrix is widely known to promote breast tumorigenesis (Xu et al., 2023). Owing to such changes in the breast tumor microenvironment, cells must adapt to the altered stiffness conditions. This, in turn, supports cancer cell growth, metabolism and migration, which drives tumorigenesis (Ishihara and Haga, 2022; Deng et al., 2022; Wu et al., 2023). MDA-MB-231 and MCF7 cells differ in their mechanical properties and invasive behavior (Aermes et al., 2021), making them an attractive model for studying the role and regulation of cellular mechanosensing. This is partly mediated by their differential regulation of the actin cytoskeleton (Ansardamavandi et al., 2018). Although differential Golgi organization in MDA-MB-231 and MCF7 cells is well known, its possible relevance and role in their distinct mechano-responsiveness remains largely unexplored. Thus, despite cell–matrix adhesion being known to regulate Golgi organization and function (Singh et al., 2018), only one study so far has evaluated the mechanosensitive nature of the Golgi (Théry and Pende, 2019). Our earlier studies have shown adhesion-dependent Arf1 activation to be vital for Golgi regulation (Singh et al., 2018). Changes in extracellular mechanical cues affect Golgi rheology, leading to lipin-1 inactivation and alterations of diacylglycerol levels at the Golgi (Romani et al., 2019). This is also mediated by Arf1-dependent regulation of transcription factors like SREBP1 and SREBP2 (Romani et al., 2019). Studies have shown elevated Arf1 levels and activation to play a key role in the hyper-proliferative behaviors of breast cancer cells (Boulay et al., 2011; Boulay et al., 2008). Our independent studies evaluating the differential expression of Golgi-associated genes in MDA-MB-231 versus MCF7 cells identified AXL as one of the top regulatory candidates, revealing an AXL-Arf1-Golgi regulatory pathway (Joshi et al., 2025 preprint).

In adherent MDA-MB-231 cells, Golgi organization responds to changing matrix stiffness, becoming more organized as stiffness increases, revealing a novel stiffness-dependent regulatory control of the Golgi. In contrast, in MCF7 cells, the disorganized Golgi is unaffected by changing matrix stiffness. This is despite both cell lines exhibiting stiffness-dependent increase in spreading. These differences likely arise from changes in the expression of Golgi-associated AXL, which regulates their distinct mechanosensory response in breast cancer cells.

These findings reveal that Golgi organization and its regulation by matrix adhesion differ between subtypes. In MDA-MB-231 cells, a mesenchymal, invasive TNBC model, the Golgi organization responds to adhesion and stiffness, supporting functions like cell-surface glycosylation and tubulin acetylation. Known for their high plasticity and dynamic ECM responsiveness (Riehl et al., 2021; Guo et al., 2020; Li et al., 2016), these cells likely exhibit significant Golgi changes in response to adhesion cues, reflecting their reliance on cytoskeletal remodeling (Schlienger et al., 2015) and trafficking (Zahn and Lundin-Schiller, 2024) for adhesion-dependent functions. In contrast, MCF7 cells, a luminal, epithelial-like subtype with limited invasiveness, show no stiffness-dependent Golgi changes, consistent with their reported reduced actin cytoskeletal adaptability (Gayan et al., 2017).

The conserved heterogeneity in Golgi organization at each stiffness might influence how cell populations respond to their environment. This suggests that Golgi phenotypes are neither inherently beneficial nor detrimental but reflect regulatory and functional variations. Despite differences in Golgi organization and mechano-responsiveness, MDA-MB-231 and MCF7 cells remain anchorage-independent, highlighting the need to understand differential Golgi regulation and its role in functional diversity among cancer cells.

Loss of AXL protein or its inhibition by R428 markedly disrupts Golgi organization and its stiffness responsiveness, supporting the idea that AXL has a role in mechanosensitive regulation of Golgi organization. In MDA-MB-231 cells, both AXL and Arf1 expression respond to matrix stiffness, and similar patterns are observed when AXL is expressed in MCF7 cells. The regulatory crosstalk between YAP (YAP1) and AXL might mediate this effect. In head and neck squamous cell carcinoma, YAP overexpression promotes AXL expression (Li et al., 2020), and AXL also regulates YAP and its oncogenic activity (Xu et al., 2011). The effect of AXL on Arf1 expression might also be mediated through its regulation of YAP in these cells (Xu et al., 2011; Ghiso et al., 2017). Matrix stiffness-dependent AXL and Arf1 regulation might affect more than the Golgi organization and functions in breast cancer. Whether this crosstalk occurs in other cells or is breast cancer-specific remains unknown.

AXL-mediated regulation of Arf1 activation, normalized for change in Arf1 expression, suggests this to be an additional element to their crosstalk. Our independent studies also show that inhibiting AXL or Arf1 in MDA-MB-231 cells displaces them from the Golgi, underscoring the role of their localization in maintaining Golgi organization (Joshi et al., 2025 preprint). This suggests their regulation of each other's activation could also impact their localization and function (Joshi et al., 2025 preprint). Although Haines et al. (2015) have reported a modest increase in Arf1 activation upon AXL inhibition (Haines et al., 2015), our findings in both MDA-MB-231 and AXL-expressing MCF7 cells confirm and extend this crosstalk by demonstrating that AXL actively promotes stiffness-dependent changes in Arf1 levels and activation. This regulation, in turn, impacts Golgi organization and function, adding new insights into the mechanosensitive interplay between AXL and Arf1.

The high-affinity AXL ligand Gas6 mediates signaling involved in regulation of tumor growth, metastasis, invasion, EMT, angiogenesis, drug resistance, immune regulation and stem cell maintenance (Tanaka and Siemann, 2021). Understanding how AXL-Gas6 signaling at the plasma membrane influences the role of AXL at the Golgi could deepen insights into the AXL-Arf1-Golgi crosstalk. Our ongoing work is exploring this.

On soft matrices (0.5 and 2.5 kPa), MCF7 (AXL-negative) and AXL-MCF7 cells show strikingly different Golgi phenotypes, with a distinct diffuse pattern of the cis-Golgi marker GM130, indicating significant Golgi disorganization. Challenges in imaging cells on softer gels made characterizing this phenotype difficult. The minimal change in AXL and Arf1 expression between 0.5 and 2.5 kPa suggests their levels are not strongly regulated within this stiffness range, implying other factors might control Golgi organization in MCF7 cells at lower stiffness. This is also distinctly different from MDA-MB-231 cells, where even in the absence of AXL (siRNA knockdown), the Golgi does not have a diffused organization at lower matrix stiffness. This suggests there could be more to the mechanosensitive regulation of the Golgi in breast cancer cells, particularly in the case of MCF7 cells.

AXL expression and its inhibition modulate stiffness-dependent cell spreading in breast cancer cells. Without AXL, cells with a disorganized Golgi exhibit increased spreading across all stiffness conditions. AXL is also known to regulate focal adhesion dynamics (Abu-Thuraia et al., 2020), which could be responsible for these changes observed. Golgi organization, through its role in regulating microtubule-dependent trafficking, could influence focal adhesion dynamics (Rong et al., 2021). Golgi-associated microtubules interact with focal adhesions to regulate cell polarization and durotaxis on substrates of increasing stiffness (Rong et al., 2021). Changes in tubulin acetylation could be mediated through the regulation of Arf1 levels or activation (Zhang et al., 2024) and Golgi organization (Chabin-Brion et al., 2001), both regulated by changing matrix stiffness and dependent on AXL expression and/or activation. Our findings show that AXL and Arf1 act via a common pathway to regulate matrix stiffness-dependent Golgi organization, highlighting their mechanistic link. Increased GBF1–Golgi colocalization and Arf1 activation in AXL-MCF7 cells compared to in MCF7 cells highlights the crucial role of the AXL-Arf1 axis in maintaining Golgi organization.

Studies have implicated alterations in cell surface glycan signatures as key mediators of breast cancer tumorigenesis (Scott and Drake, 2019). As tumors become more metastatic and invasive, specific N- and O-glycan changes are observed on glycosylated proteins and lipids trafficked through the Golgi to the plasma membrane (Thomas et al., 2021). These changes in glycan signatures are strongly linked with alterations in Golgi organization and function (Pothukuchi et al., 2019). Studies have also hinted towards the possibility of matrix stiffness affecting the surface glycans of cells called the glycocalyx (Mahmoud et al., 2021), which could also be affected by Golgi organization and function. In our studies, individual and combined targeting of AXL and Arf1, each causing comparable Golgi disorganization, led to increased cell surface WGA and ConA lectin staining in MDA-MB-231 cells. We believe this reflects a direct functional consequence of the observed Golgi disorganization.

Multiple studies have shown that elevated sialic acids, GlcNA and high-mannose glycans, as detected by WGA and ConA lectins, are closely linked to malignant traits like invasiveness, metastasis and immune modulation in breast cancer cells (Häuselmann and Borsig, 2014; De Leoz et al., 2011; Ščupáková et al., 2021). Changes in these glycan modifications can affect receptor stability, localization, clustering and signaling, all contributing to malignant behavior (Schultz et al., 2012; Stowell et al., 2015; Wu et al., 2022; Kariya et al., 2017). Glycosylation changes, such as increased sialylation and N-glycan branching, can directly modulate integrin clustering and function, promoting cell adhesion, migration, and invasion – key drivers of cancer metastasis (Huang et al., 2021; Zhao et al., 2008; Kariya et al., 2017; Ata and Antonescu, 2017). Sialylation and GlcNAc branching also enhance EGFR stability and signaling, potentially influencing tumor progression and therapeutic resistance (Ankenbauer et al., 2023; Li et al., 2014; Britain et al., 2018). How differential Golgi organization and glycosylation affect receptor functions across various different matrix stiffness could further illuminate the broader impact of this regulatory pathway on cancer cell behavior.

Breast cancer aggressiveness is closely linked to increased ECM stiffness, which enhances invasive and metastatic potential (Xu et al., 2023). Our findings suggest that the AXL-Arf1-Golgi pathway functions as a key mechanosensitive axis, regulating changes in Golgi organization and function with potential implications for matrix stiffness-driven tumor progression in breast cancer.

## MATERIALS AND METHODS

### Antibodies and reagents for western blotting, immunostaining and lectin staining

Primary antibodies were diluted in 5% BSA in 1× Tris-buffer saline with 0.1% Tween 20 (TBST) and 1× PBS and used for western blotting and immunostaining. Antibodies used were against: pAkt S473, cat. no. #4060S (1:2000); Akt, cat. no. 9272S (1:1000); AXL (C89E7), cat. no. 8661S (1:2000) and phospho-Axl (Tyr702) (D12B2) (Y702), cat. no. 5724S (1:1000) [all purchased from Cell Signaling Technology (CST)]; GAPDH, cat. no. #9545 (1:5000) (Sigma); GM130 clone 35, cat. no. 612008 (1:100) (BD Transduction); ARF1 monoclonal, clone 1D9, cat. no. MA3-060 (1:500) (Invitrogen); β-tubulin, clone E7, cat. no. AB_2315513 (1:1000) [Developmental Studies Hybridoma Bank (DSHB)]; anti-acetylated tubulin, Mouse, cat. no. T7451 (1:2000) (Sigma); ARF1, cat. no. ab183576 (1:3000) and anti-GBF1 antibody #cat. no. ab86071 (1:2000) (both from Abcam); and ubiquitin/poly-ubiquitin (P4D1), cat. no. sc-8017 (Santa Cruz Biotechlogy) (1:1000) [generously provided by Prof. Manas Kumar Santra (BRIC-NCCS, Pune)]. Fluorophore-conjugated lectin probes were purchased from Invitrogen Molecular Probes: ConA–Alexa Fluor 488 (cat. no. C11252), WGA–Alexa Fluor 488 (cat. no. W11261). Phalloidin–Alexa Fluor 488, cat. no. A12379 (1:500) was purchased from Invitrogen. DAPI, cat no. D1306 (0.05 mg/ml stock diluted to 1:50 times) was purchased from Invitrogen. Secondary fluorescent conjugated antibodies (to Alexa Fluor 488 and Alexa Fluor 568) were purchased from Invitrogen Molecular Probes and used at a dilution of 1:1000. HRP-conjugated secondary antibodies were purchased from Jackson ImmunoResearch and used at a dilution of 1:5000 in 2.5% milk made in 1× TBST. RIPA buffer used for cell lysis was composed of the following chemicals: 50 mM Tris-HCl pH 7.5, 0.5% sodium deoxycholate, 0.05% SDS, 150 mM EDTA, 1% NP40 and 150 mM NaCl. Before lysis using the buffer, the following components were freshly added: protease inhibitor cocktail (PIC, Roche, cat. no. 04693116001), 1 mM sodium fluoride and 1 mM sodium orthovanadate.

### Reagents for 2D polyacrylamide hydrogels

Acrylamide (HiMedia, Chennai, India, cat. no. MB068), bisacrylamide (HiMedia, cat. no. MB005), APS, TEMED (Sigma, cat. no. T9281), MES buffer (Sigma, cat. no. M8250), NHS (Sigma, cat. no. 130672), EDC (Sigma, cat. no. E1769), Toluene (Qualigens, Los Angeles, CA, USA, cat. no. 32507), Silane (Sigma, cat. no. 440159), Fluoromount-G (Southern Biotech, Birmingham, AL, USA, 0100-01), and collagen I, rat tail (Gibco, Thermo Fisher Scientific, Waltham, MA, USA, cat. no. A1048301) were used.

### Reagents for AXL, Arf1, Akt and proteasome inhibition studies

AXL kinase inhibitor-R428 (Bemcentinib and BGB324), cat. no. 21523, was purchased from Cayman Chemical (USA). Golgicide-A (GCA), cat. no. G0923, was purchased from Sigma. Akt Inhibitor VIII, A3153-100MG (TCI Chemicals) was generously provided by Dr Mayurika Lahiri (IISER Pune). MG132 proteasome inhibitor cat. no. HY-13259 (MedChemExpress) was generously provided by Prof. Kundan Sengupta (IISER Pune).

### Plasmids and oligonucleotides

GalTase-RFP (GalT–RFP) and Mannosidase-II-GFP (ManII–GFP) constructs were obtained from Prof. Jennifer Lippincott Schwartz (HHMI, USA). The ABD–GFP construct was obtained from Prof. Satyajit Mayor (NCBS Bangalore, India); this construct encodes the Arf-binding domain (ABD) of ARHGAP10 fused to GFP. This domain binds explicitly to the GTP-bound (active) form of Arf1 and is used as a biosensor for monitoring active Arf1 localization within cells (Kumari and Mayor, 2008; Rajeshwari et al., 2023). The peGFP-N1-AXL plasmid construct was obtained from Dr. Stéphane Bodin (CRMB CNRS). pVSV-G (Addgene #138479), gag/pol (Addgene #14887), AXL-pBABE (Addgene #105936), pBABE-puro (Addgene #1764), and pMIG-w (Addgene #12282) plasmid constructs were purchased from Addgene.

siRNA against human AXL sequence (siAXL) was designed and purchased from Merck (Sigma). The siRNA sequence used was described previously (Li et al., 2009; Holland et al., 2005; forward, 5′-GAAAGAAGGAGACCCGTTA-3′ and reverse, 5′-TAACGGGTCTCCTTCTTTC-3′). siRNA against the human Arf1 sequence (siArf1) was designed and purchased from Merck (Sigma). The siRNA sequence used was described earlier (Boulay et al., 2008; forward, 5′-ACAGCAAUGACAGAGAGCGUGUGAA-3′ and reverse, 5′-TTCUCUCGCTCTCTGTCUTTGCTGT-3′).

### Cell culture and transfections

MDA-MB-231 was obtained from the European Collection of Cell Cultures (ECACC). The MCF7 cell line (ATCC) was obtained from Prof. Sanjay Gupta at ACTREC (TMC), Navi Mumbai, India. All cell lines were cultured using Gibco DMEM from Thermo Fisher Scientific, adding 10% pen-strep (Penicillin-Streptomycin from Gibco by Life Technologies; Thermo Fisher Scientific, cat. no. 15140122) and 5% fetal bovine serum (FBS; Gibco, cat. no.,10270106). 0.05% trypsin was used to detach cells, and an excess of culture medium was used to neutralize the action of trypsin. The MCF10A cell line was obtained from Dr Madhura Kulkarni's lab at CTCR, IISER Pune & Prashanti Cancer Care Mission (PCCM), and cultured using Gibco DMEM/F12 media (cat. no. 12500062) with the addition of 5% horse serum (Invitrogen, #16050-122), 10% Pen-strep, 20 ng/ml EGF (Sigma, cat. no. E9644), 0.5 mg/ml hydrocortisone (Sigma, cat. no. H0888-5G), 100 ng/ml cholera toxin (Sigma, cat. no. C8052-1MG) and 10 μg/ml insulin (Sigma, cat. no. I1882-100MG). The project and grant supporting it have BSL2 clearance from the Institutional Biosafety Committee (IBSC).

For transfection studies, cells were seeded in 6 cm dishes to attain a confluency of 60% and allowed to attach and spread for 5 h. Using Gibco OptiMEM medium and transfection agents polyethylenimine (PEI; Sigma) and Lipofectamine 2000 (Thermo Fisher Scientific) (for MCF7 and MDA-MB-231 cells, respectively), the transfection mix was prepared and kept at room temperature for 30 min before adding to the cells seeded in 6 cm dishes. The medium in Lipofectamine 2000-transfected dishes was changed 12 h post-transfection, and cells were used for experiments 36 h post transfection.

### Preparation of 2D polyacrylamide hydrogels of various stiffnesses

Polyacrylamide gels of various stiffnesses were prepared by cross-linking 40% polyacrylamide and 2% bis-acrylamide. Briefly, gels were prepared between a glass slide uniformly coated with a hydrophobic layer of nail paint, and 12-mm glass coverslips were activated using toluene/silane solution (9:1 ratio). Gel thickness was kept constant by using equal volumes for all gels. After polymerization, gels attached to coverslips were removed, washed with PBS, treated with 0.1 M N-hydroxysuccinimide (NHS) plus 0.2 M 1-ethyl-3-(3-dimethylaminopropyl)carbodiimide (EDC) (as a crosslinker), and coated with 25 μg/ml collagen overnight at 4°C. Gels were treated with UV cycles in tissue culture hood and equilibrated in DMEM before plating cells. 10,000 and 5×10^5^ MDA-MB-231, MCF7 and AXL-MCF7 cells were seeded per gel for cell spreading and western blotting studies, respectively. Cells were allowed to spread for 24 h and processed thereafter. For cell spreading studies, cells were fixed with 3.5% paraformaldehyde for 15 mins, stained with phalloidin at 4°C overnight, and followed by DAPI before mounting on glass slides. For western blotting, cells on each coverslip were lysed on ice with 80 μl of RIPA buffer plus protease (1× PIC) and phosphatase (NaF and sodium orthovanadate) inhibitors. Samples were protein-estimated using Pierce™ BCA Protein Assay Kit, cat no. #23225, and 30 μg total protein was used for western blotting.

### Inhibitor studies

Post 24 h of cell seeding on 2D hydrogels or glass coverslips, R428 treatment was performed on cells for 12 h at 1 µM concentration. R428 was reconstituted in DMSO, and hence, a comparable volume of DMSO was added to control cells. 24 h after seeding on 2D PA-gels and glass coverslips, cells were treated with GolgicideA (GCA) for 24 h at 1 µM concentration. Comparable volume of DMSO was added to control cells. When dual (R428 plus GCA) inhibitor treatments were done, cells were incubated with GCA (1 µM) for 24 h. R428 (1 µM) was added to cells after 12 h of GCA treatment, with an appropriate volume of DMSO added to control cells. 24 h after seeding, cells were treated with Akt inhibitor VIII (reconstituted in DMSO) (1 µM) for 5 h, with appropriate DMSO control. MG132 inhibitor (10 µM) treatment was initiated when cells were seeded on collagen-coated gel or glass for 24 h with proper DMSO control.

### AXL and Arf1 knockdown using siRNA oligonucleotides

After 24 h of MDA-MB-231 cell seeding, two consecutive transfection shots were performed using Lipofectamine RNAiMAX for the oligonucleotides siAXL and siArf1 (50 and 100 pmol siRNA were used, respectively). The first shot was after 24 h of seeding, and the second one after 24 h of the first transfection shot. For dual knockdowns (siAXL+siArf1), the same siRNA concentrations (50 pmol for siAXL and 100 pmol for siArf1) were simultaneously used, and the transfection protocol was identical to that used for individual knockdowns. Then, cells were allowed to stay with medium containing the transfection mix for another 48 h. After 48 h, cells were harvested and seeded on the collagen-coated 2D hydrogels or glass and were incubated for 24 h in 5% CO_2_. The remaining volume of cell suspension (approximately the same cell number) was plated on 60 mm cell culture dishes for preparing lysates (post 24 h of replating) to validate the efficiency of the AXL, Arf1 and dual knockdowns (siAXL+siArf1) using western blotting.

### SDS-PAGE and western blotting

Protein lysates (normalized to total protein or cell equivalents) were resolved by SDS-PAGE and transferred onto PVDF membranes. Membranes were blocked with 5% skimmed milk in TBST (0.1% Tween-20 in TBS) for 1 h at room temperature (RT), followed by overnight incubation at 4°C with primary antibodies diluted in 5% BSA in TBST. After three washes with TBST, membranes were incubated at RT with HRP-conjugated secondary antibodies diluted in 2.5% skimmed milk in TBST. Following additional washes, chemiluminescence signal was obtained using Immobilon substrate (diluted in TBST when required) and visualized with a GE Healthcare LAS4000 chemiluminescent imager. Band intensities were quantified using ImageJ (NIH). Blot transparency data is shown in Fig. S8.

### Immunofluorescence staining of cells for the Golgi on 2D hydrogels and glass

For immunofluorescence assays, cells fixed with 3.5% paraformaldehyde (PFA) were incubated in permeabilization buffer [Triton X-100 (0.05%) diluted in 5% BSA and made in 1× PBS] for 15 min at room temperature. Post permeabilization, two washes were given in 1× PBS, followed by blocking with 5% BSA at room temperature for 1 h. Three washes were given after blocking. Samples were incubated with primary antibody overnight at 4°C. Three washes were given post-incubation with primary antibody followed by a 1-h incubation with secondary fluorescent antibody at room temperature. Samples were given three washes and then mounted on slides using Fluoromount-G (Southern Biotech), Cat no. #0100-01. Slides were maintained at room temperature under dark conditions to dry, then moved to 4°C until confocal imaging.

### Determining the Golgi distribution profile in a cell population

MDA-MB-231 cells adherent on gels of varying stiffness (0.5 kPa, 2.5 kPa, 23 kPa, glass) spread variably, with differential predominant Golgi organization. These cells stained for GM130 (cis-Golgi marker) were visually characterized as being distinctly ‘organized’ or ‘disorganized’ followed by capturing confocal cross-section images. These images were thresholded at comparable settings using ImageJ FIJI. The number of Golgi objects detected in cells containing ‘organized’ Golgi at 0.5 kPa, 2.5 kPa and 23 kPa and on glass was plotted and compared to the number of cells containing ‘disorganized’ Golgi. This revealed the ‘organized’ Golgi to have 10 or fewer object numbers. This was significantly more in cells with a ‘disorganized’ Golgi. Using the same threshold settings applied for characterizing Golgi organization in MDA-MB-231 cells, we also analyzed MCF7 and AXL-MCF7 (and AXL–GFP transfected) cells to compare object counts between their predominant disorganized and organized Golgi phenotypes, respectively. By establishing this strong object count association in cells on gels as well as glass, we could use our visual classification of these cells to determine their distribution profile in cell populations.

During the distribution profile assessment, cells were categorized as having: (1) organized Golgi – compact, perinuclear Golgi with few or no distinct puncta; (2) disorganized Golgi – loss of compactness with multiple discrete Golgi objects dispersed around the perinuclear region and cytosol; or (3) diffused Golgi – lack of perinuclear clustering with Golgi elements appearing dispersed or diffused throughout the cytoplasm. The number of cells in each group was then used to calculate the distribution of organized versus disorganized Golgi (in percent) in each population for a given time point or treatment. Data from multiple experiments using these percentage values were plotted accordingly. Categorization was done by the authors who were aware of the experimental conditions and verfied by other authors who were unaware.

### Lectin staining of cells on 23 kPa hydrogel and glass

MDA-MB-231 cells (10^4^) were seeded on 23 kPa polyacrylamide (PA) hydrogels or glass coverslips coated with 25 μg/ml collagen-I and incubated for 24 h. To prevent cold shock, cells were equilibrated at room temperature for 5 min, followed by 10 min at 4°C. After two washes with cold 1× PBS, cells were incubated on ice for 10 min in the dark with WGA–Alexa Fluor 488 (1 ng/μl) or ConA–Alexa Fluor 488 (50 ng/μl). Cells were washed with cold 1× PBS, fixed with 3.5% PFA for 15 min at room temperature, rewashed, mounted, and air-dried in the dark. Cross-section images were acquired using a Zeiss LSM 780 confocal microscope (40× oil objective). Integrated density per cell was quantified using manual thresholding of cell images in ImageJ/Fiji.

### Microscopy imaging for cell spreading analysis

Cross-section images for phalloidin-stained cells were captured using a 20× air objective on the EVOS FL Auto Imaging System (Thermo Fisher Scientific, Waltham, MA, USA, AMAFD1000). Images for multiple frames were captured to analyze at least 100 cells per independent experiment for the respective stiffness. Cell spread analysis was performed by manual thresholding using ImageJ.

### Arf1 activity assay on 2D hydrogels and glass

MCF7 and AXL-MCF7 cells (3.5×10^5^) plated on the 2D PA hydrogels or glass coated with 25 µg/ml collagen-I for 24 h, were kept on ice and lysed using the activity assay buffer (Pawar et al., 2016). For the lysis of cells, each PA hydrogel or glass-coated coverslip was incubated with 20 µl activity assay buffer for 10 min. Three such incubations of 10 min, with 20 µl activity assay buffer, were done for each gel or glass coverslip. The cells with lysis buffer were kept on ice during these treatments. At the end of 30 min (three incubations), all the lysate was collected from each well. To ensure an approximately equal amount of lysate protein was obtained from the lysis of cells on gels and coverslips, we used five 23 kPa PA hydrogels and three glass coverslips for lysis. This means we should get 5×60 µl=300 µl of lysate from the PA hydrogel and 3×60 µl=180 µl of lysate from glass coverslips. To these cell lysates, additional activity assay buffer was added to make up the total volume to 500 µl for both 23 kPa and glass. For the pulldown assay, 400 µl of these cell lysates were incubated with 60 µg of glutathione S-transferase (GST)-tagged Golgi-localized γ-ear containing Arf-binding protein 3 (GGA3) fusion protein (GST–GGA3) bound to Glutathione Sepharose™ 4B beads Cytiva (cat. no. 17-0756-01, Merck). These were incubated at 4°C on a roto-spinner at 9 rpm for 35 mins. Beads were finally spun down, washed three times with activity assay buffer, and eluted in 20 µl of 2.5× Laemmli buffer. To 80 µl of the cell lysate collected at the start, 20 µl of 5× Laemmli buffer was added, and this was used as whole cell lysate (WCL). 12.5% SDS-PAGE resolved the entire GGA3 pulldown eluate samples and 20 µl of WCL samples.

### Generation of stable AXL-expressing MCF7 cells

HEK293T cells were seeded at an appropriate density to generate retroviral particles for transduction. Post 24 h of HEK293T seeding, transfection using Lipofectamine 2000 was performed with pBABE-puro, pMIG-w and AXL-pBABE-puro plasmids along with pVSVG and pGagPol in respective dishes. Here, pMIG-w (a retroviral vector with the GFP tag) was used as a control to check the transfection efficiency in HEK293T and transduction efficiency in MCF7 cells post-addition of the viral titer. After 48 h of transfection, 1.5×10^5^ MCF7 cells were seeded for transduction with the viral titer. Also, 2 ml of 10% DMEM was added to the transfected dishes. Post 72 h of transfection, viral titer was collected and filtered using the 0.2 µm filter. This was followed by adding 3 ml of the viral titer, 1 ml 10% DMEM and 4 µl of 10 µg/ml polybrene to prior-seeded MCF7 cells (∼50% confluent). Post 72 h of viral titer addition, puromycin (final concentration, 1.6 µg/ml) selection was performed on transduced MCF7 cells. Post 72 h of selection, cell viability was checked. The cell colonies surviving the selection process were replated on 24-well plate for further culturing and expansion to confirm and check AXL protein expression by western blotting.

### Image analysis and quantitation

ImageJ FIJI was used to process images with respective scale bars for all imaging experiments. Golgi object count analysis for MDA-MB-231, MCF7, MCF7 (AXL-GFP), and AXL-MCF7 cells were performed on cross-section binary images labelled for the cis Golgi marker GM130 (Alexa Fluor 568 channel). Line plot analysis was performed using the ImageJ FIJI software to look at the overlap of markers in images. Huygens Professional software (SVI) was used to LSM files and perform colocalization analysis to look at the overlap of different markers. Colocalization analysis for AXL, ABD–GFP and GBF1 localization at the Golgi for MDA-MB-231, MCF7 and AXL-MCF7 cells was done using the Huygens Professional software (SVI) ‘Colocalization Analyzer Advanced’ tool. The threshold for each cross-section image was set using the Costes method, and the Golgi marker channel was thresholded manually for further analysis to obtain the Pearson's coefficient value using the software.

### Statistical analysis

All the statistical analysis was done using GraphPad Prism analysis software. Statistical analysis for western blotting using absolute data (not normalized to a condition or control) was done using the two-tailed unpaired Mann–Whitney *U*-test. A two-tailed single-sample unpaired *t*-test was used for western blot data, normalized to the control. Statistical analysis for the percentage distribution profile for Golgi organization was done using one-way ANOVA, a multiple comparison test, with Tukey's method for error correction. The Pearson's coefficient analysis data for colocalization of different markers was tested for statistical significance using the two-tailed unpaired Mann–Whitney *U*-test. The data for cell spread area, lectin intensity and Golgi object comparisons across different conditions/cell lines were also tested for statistical significance using a two-tailed unpaired Mann–Whitney *U*-test.

## Supplementary Material

10.1242/joces.263956_sup1 Supplementary information
