# Supplementary material for: Differential AXL expression and Arf1 regulation control stiffness-dependent Golgi organization in breast cancer cells
Source: J Cell Sci. 2026 Feb 3;139(2):jcs263956. doi: 10.1242/jcs.263956 (PMC12912268; doi:10.1242/jcs.263956)
Supplement: Supplementary information [file joces-139-263956-s1.pdf]

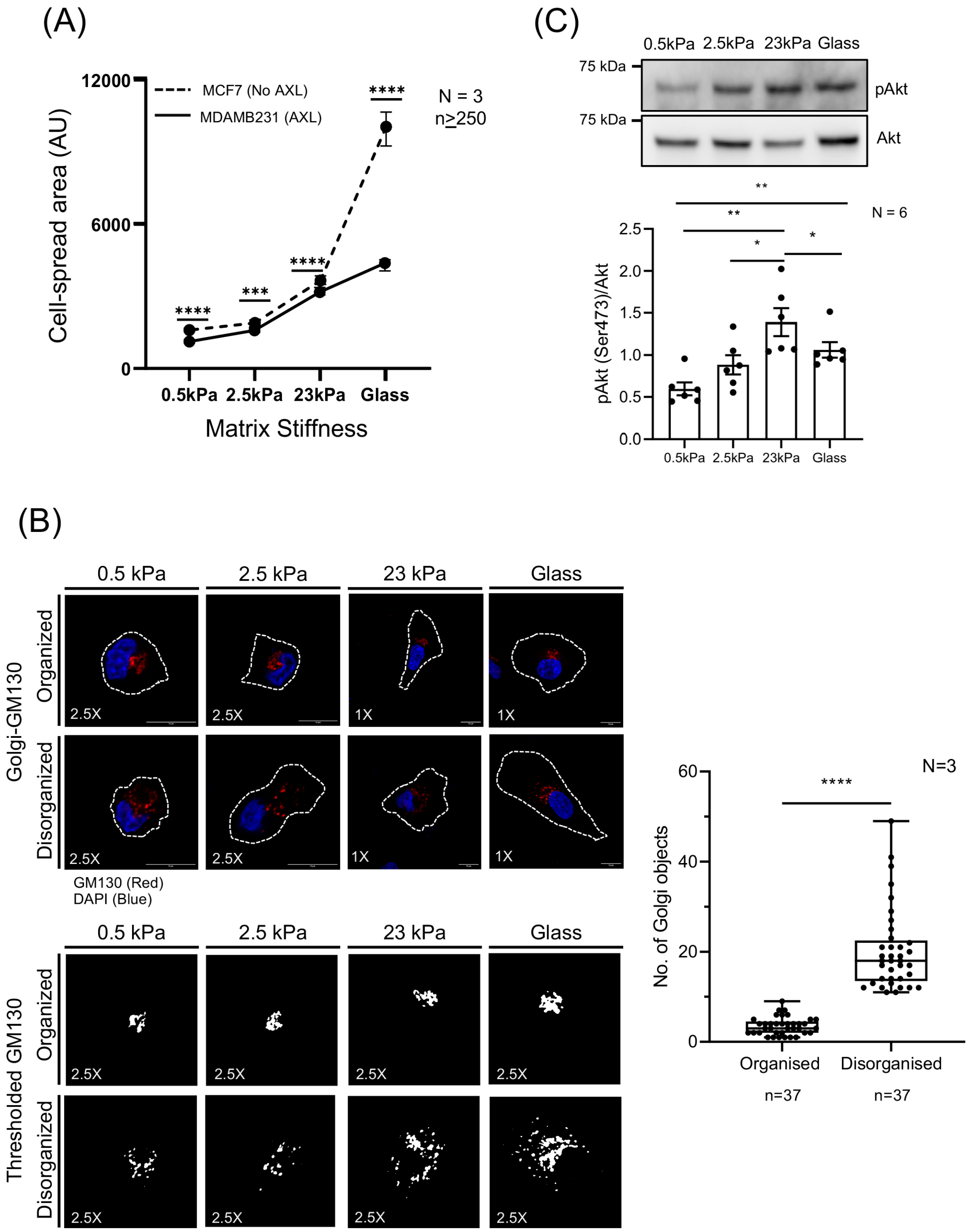

Fig. S1. (A) Cell spread area trends of MCF7 and MDAMB231 on gels of varying stiffness and glass. The graph represents the trend for cell spread area for n ≥250 cells plotted as median with 95%CI from N=3 experiments. **(B)** Cross section images of MDAMB231 cells stained for GM130 (red) and nuclei (DAPI, blue) on varying stiffness show “organized” and “disorganized” Golgi. Thresholded images (bottom) highlight Golgi objects in white. Quantification of cis-Golgi objects per cell (n=37, N=3) is shown as all data points with median ± minimum and maximum. Golgi object count quantification was performed on randomly selected control cell images with organised or disorganised Golgi phenotypes from three independent experiments, also presented in Fig. 6A. **(C)** Representative blots for Serine 473-phosphorylated Akt (pAkt) and Akt in MDAMB231 cells. Graph represents the ratio of densitometric band intensities as mean ± SEM from N=6 experiments. Statistical analysis was done using Mann-Whitney U test for cell-area, Object count, and western blot analysis. (*p≤0.05, **p ≤ 0.01, ***p ≤ 0.001, ***p ≤ 0.0001, ns=non-significant).

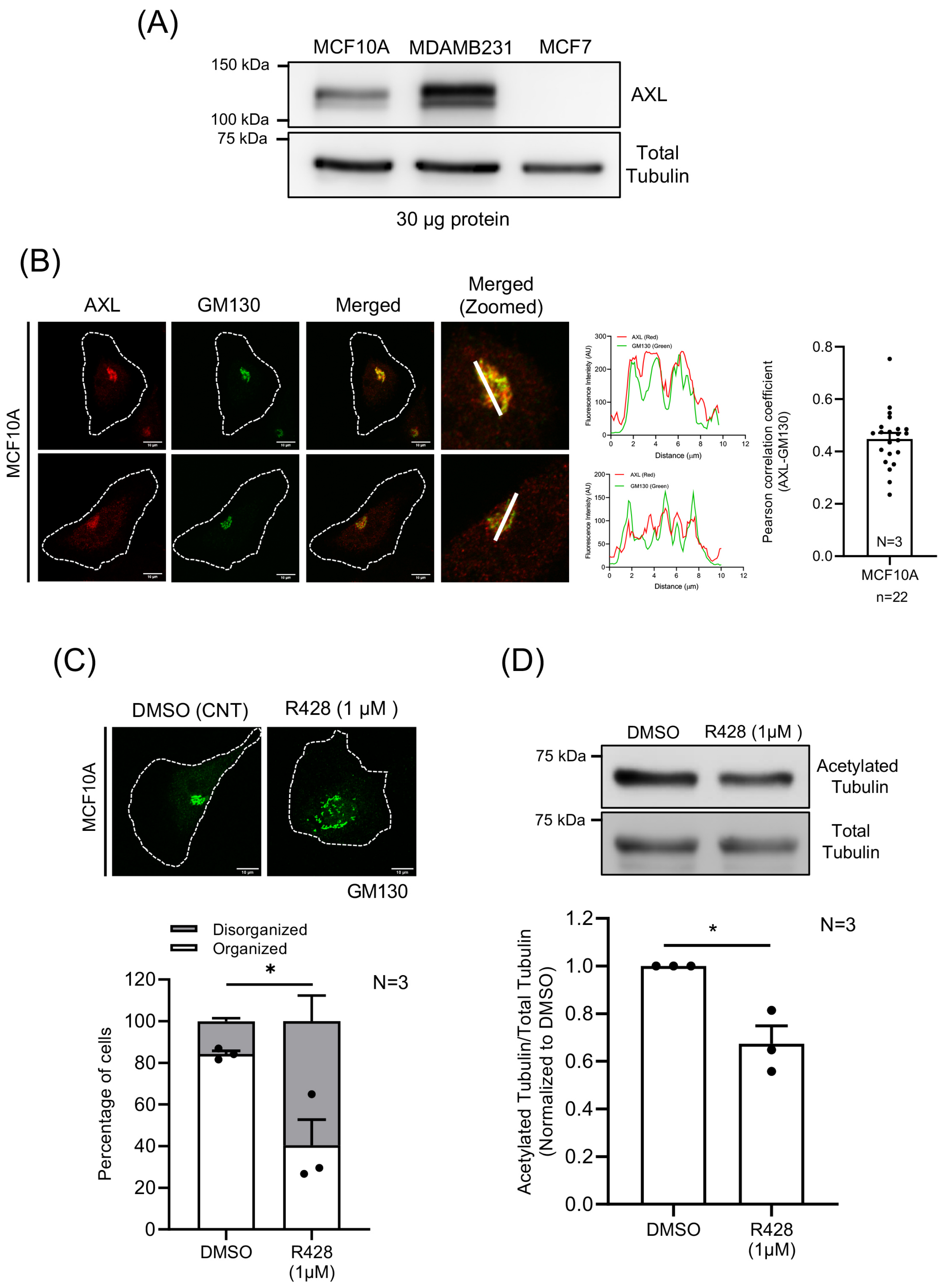
(A)
MCF10A
MDAMB231
MCF7
150 kDa
100 kDa
75 kDa
AXL
Total Tubulin
30 µg protein
(B)
AXL
GM130
Merged
Merged (Zoomed)
MCF10A
Fluorescence Intensity (AU)
Distance (µm)
AXL (Red)
GM130 (Green)
Pearson correlation coefficient (AXL-GM130)
N=3
MCF10A
n=22
(C)
DMSO (CNT)
R428 (1 µM)
MCF10A
GM130
Disorganized
Organized
Percentage of cells
*
N=3
DMSO
R428 (1µM)
(D)
DMSO
R428 (1µM)
75 kDa
75 kDa
Acetylated Tubulin
Total Tubulin
Acetylated Tubulin/Total Tubulin (Normalized to DMSO)
*
N=3
DMSO
R428 (1µM)

(E)

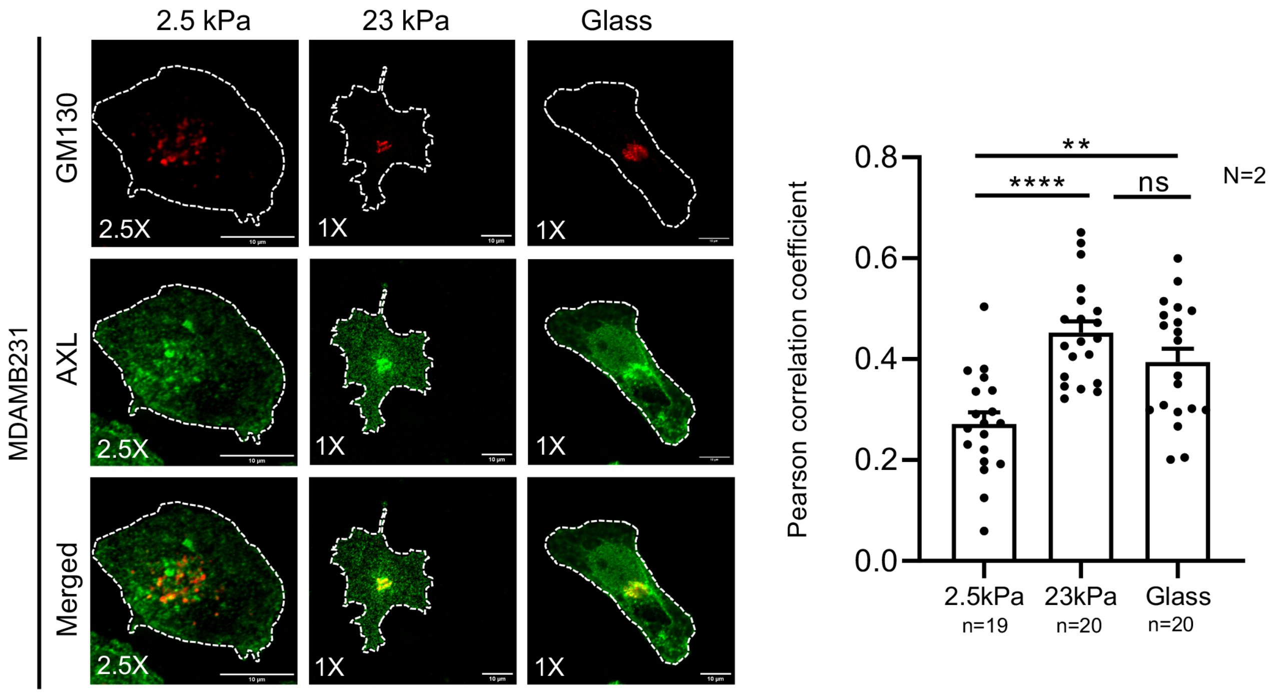
2.5 kPa
23 kPa
Glass
MDAMB231
GM130
AXL
Merged
2.5X
1X
Pearson correlation coefficient
0.0
0.2
0.4
0.6
0.8
**
****
ns
N=2
2.5kPa
23kPa
Glass
n=19
n=20
n=20

(F)

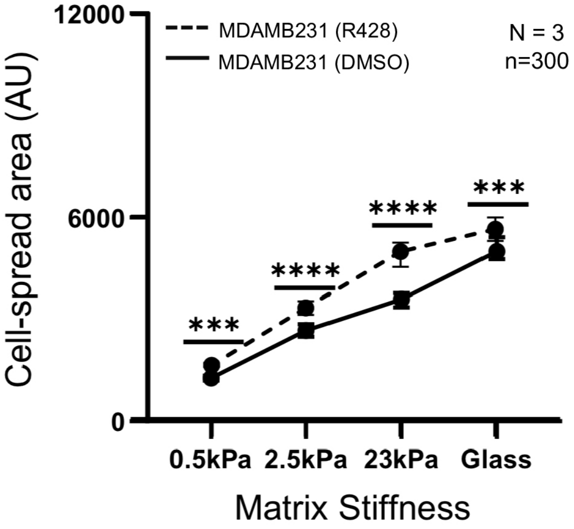
MDAMB231 (R428)
MDAMB231 (DMSO)
N = 3
n=300
Cell-spread area (AU)
0
6000
12000
***
****
****
***
0.5kPa
2.5kPa
23kPa
Glass
Matrix Stiffness

(G)

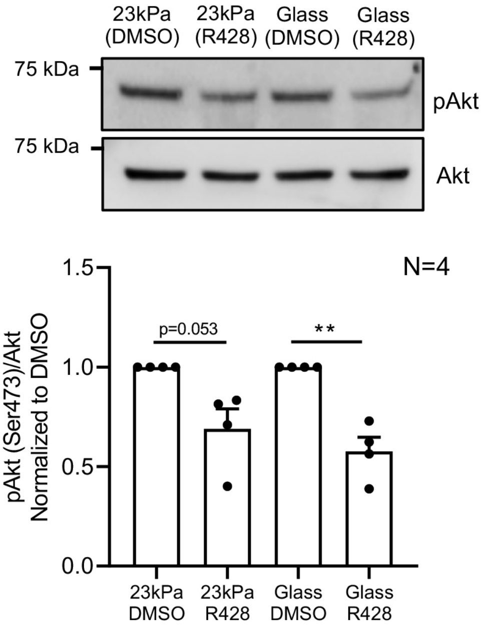
23kPa (DMSO)
23kPa (R428)
Glass (DMSO)
Glass (R428)
75 kDa
pAkt
75 kDa
Akt
N=4
pAkt (Ser473)/Akt Normalized to DMSO
0.0
0.5
1.0
1.5
p=0.053
**
23kPa DMSO
23kPa R428
Glass DMSO
Glass R428

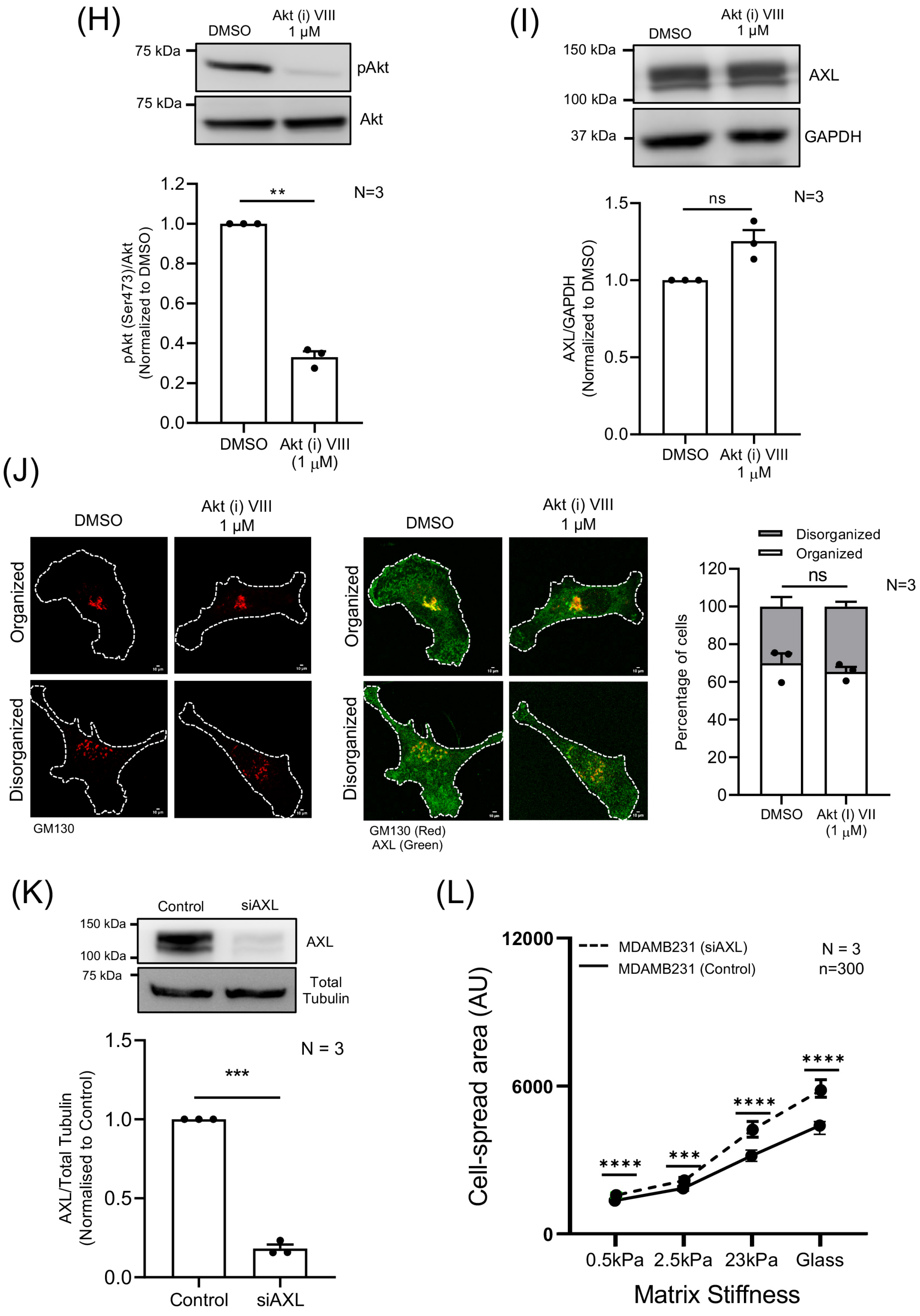

Journal of Cell Science • Supplementary information

Fig. S2. (A) Representative blots for AXL and total-tubulin in 30 μg protein in cell lysates from MCF10A, MDAMB231 and MCF7 cells **(B)** Cross-section images and intensity line plots for MCF10A immunostained for GM130 (green) and AXL (red) on glass. Graph represents the Pearson's correlation coefficients for colocalization of AXL (green) and GM130 (red) plotted as mean ± SEM from N=3 experiments **(C)** Cross-section images for predominant Golgi organization phenotype for DMSO and R428 (1μM) treated MCF10A cells immunostained for GM130. Percentage distribution profile of cells with organized and disorganized Golgi represented as mean ± SEM from N=3 experiments **(D)** Representative blots for acetylated-tubulin and total-tubulin in DMSO and R428 (1 μM) treated MCF10A cells. Graph represents the ratio of densitometric band intensities normalised to DMSO as mean ± SEM from N=3 experiments **(E)** Cross-section images of MDAMB231 cells immunostained for GM130 (red) and AXL (green) on gels of 2.5kPa, 23kPa and glass. Imaging was done at 2.5X zoom for cells at 2.5 kPa gel for better clarity. Graph represents the Pearson's correlation coefficients for AXL (green) and GM130 (red) colocalization plotted as mean ± SEM for n ≥15 cells from two N=2 experiments. **(F)** Cell spread area trend of DMSO and R428 treated MDAMB231 cells on gels of varying stiffness and glass. The graph represents the trend for cell area of n=300 cells as median and 95% CI from N=3 experiments. **(G)** Representative blots of phospho-Akt (Ser473) and Akt in DMSO and R428 treated MDAMB231 cells on 23 kPa gel and glass. Graph represents the ratio of densitometric band intensities normalised to controls as mean ± SEM from N=4 experiments. **(H, I)** Representative blots for **(H)** phospho-Akt (Ser473) and Akt and **(I)** AXL and GAPDH in DMSO and Akt VIII inhibitor (1 μM) treated MDAMB231 cells. Graph represents the ratio of densitometric band intensities **(H)** (pAkt/Akt) and **(I)** (AXL/GAPDH) normalised to control as mean ± SEM from N=3 experiments **(J)** Cross-section images of DMSO and Akt VIII inhibitor treated MDAMB231 cells on glass, with organized and disorganized Golgi immunostained for GM130 (red) and AXL (green). Percentage distribution profile of cells with organized and disorganized Golgi is represented as mean ± SEM from N=3 experiments. **(K)** Representative blots for AXL and total-tubulin in control and AXL knockdown (siAXL) MDAMB231 cells. Graph represents the ratio of densitometric band intensities (normalised to control) as mean ± SEM from N=3 experiments. The AXL and total tubulin blots are from the same experiment also presented in Fig. S7A. **(L)** Cell spread area trend of control and AXL knockdown (siAXL) MDAMB231 cells on gels of varying stiffness and glass. Graph represents the trend for cell area for n=300 cells plotted as median and 95% CI from N=3 experiments. Statistical analysis was done using Single sample Wilcoxon t test for western blots normalised (with respect to control), one-way ANOVA multiple comparisons test with Tukey's method for error correction for distribution profiles and Mann-Whitney U test for colocalization analysis. (*p≤0.05, **p ≤ 0.01, ***p ≤ 0.001, ****p ≤ 0.0001, ns=non-significant and p-values when indicated).

Journal of Cell Science • Supplementary information

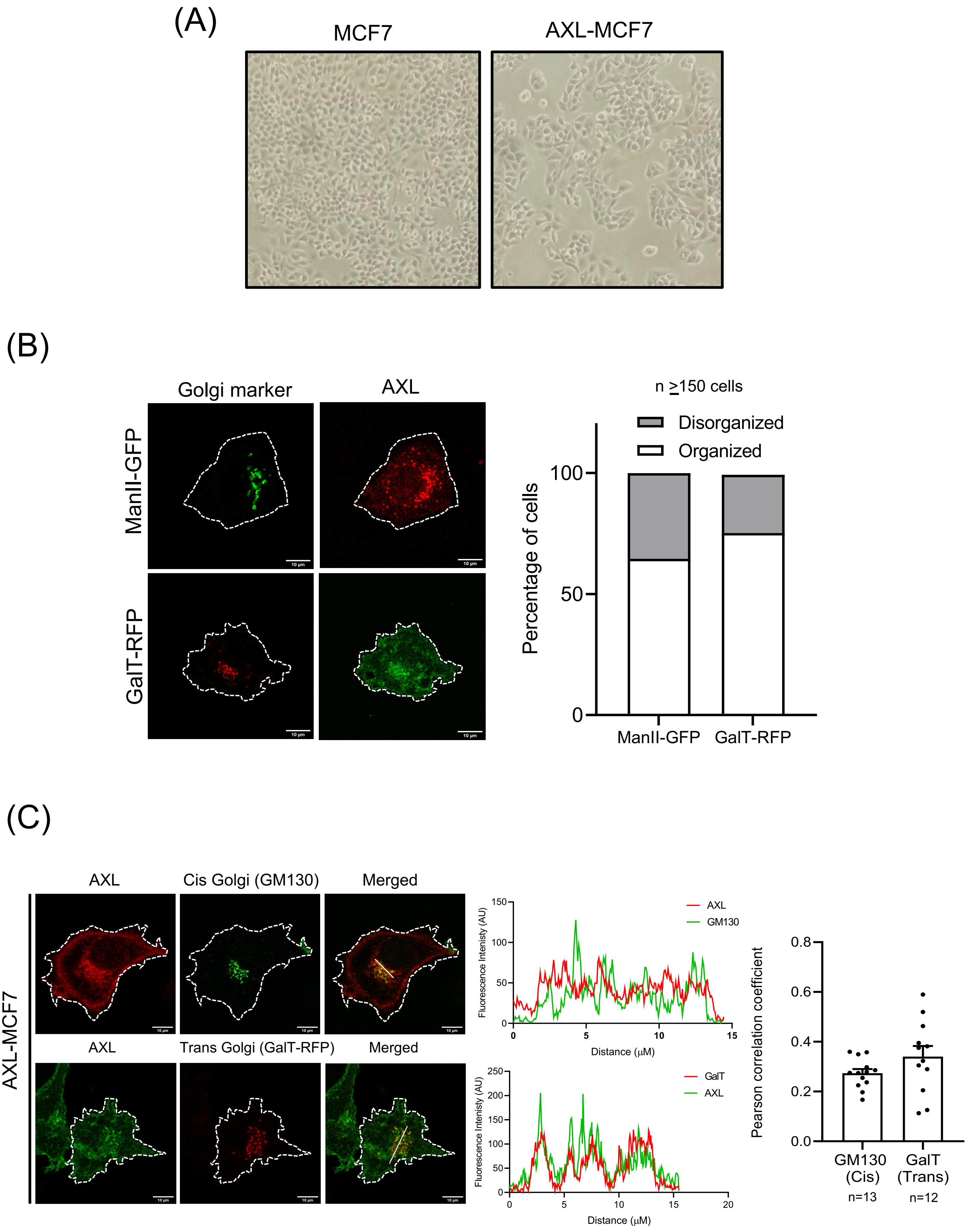

Journal of Cell Science • Supplementary information

Fig. S3. (A) Brightfield images to show cell morphology of MCF7 and stable AXL-MCF7 cells on glass **(B)** Cross-section images of AXL-MCF7 cells expressing cis-medial Golgi (ManII-GFP) and trans-Golgi (GalTase-RFP) marker and immunostained for AXL. Percentage distribution profile for cells with organized and disorganized Golgi from for n≥150 cells **(C)** Cross-section images and intensity line plots for AXL-MCF7 cells immunostained for AXL and cis-Golgi marker (GM130) and expressing trans-Golgi marker (GalTase-RFP). Graph represents the Pearson's correlation coefficients for colocalization of AXL and Golgi markers (Cis-Golgi and Trans-Golgi) plotted as mean ± SEM for n=13 and n=12 cells

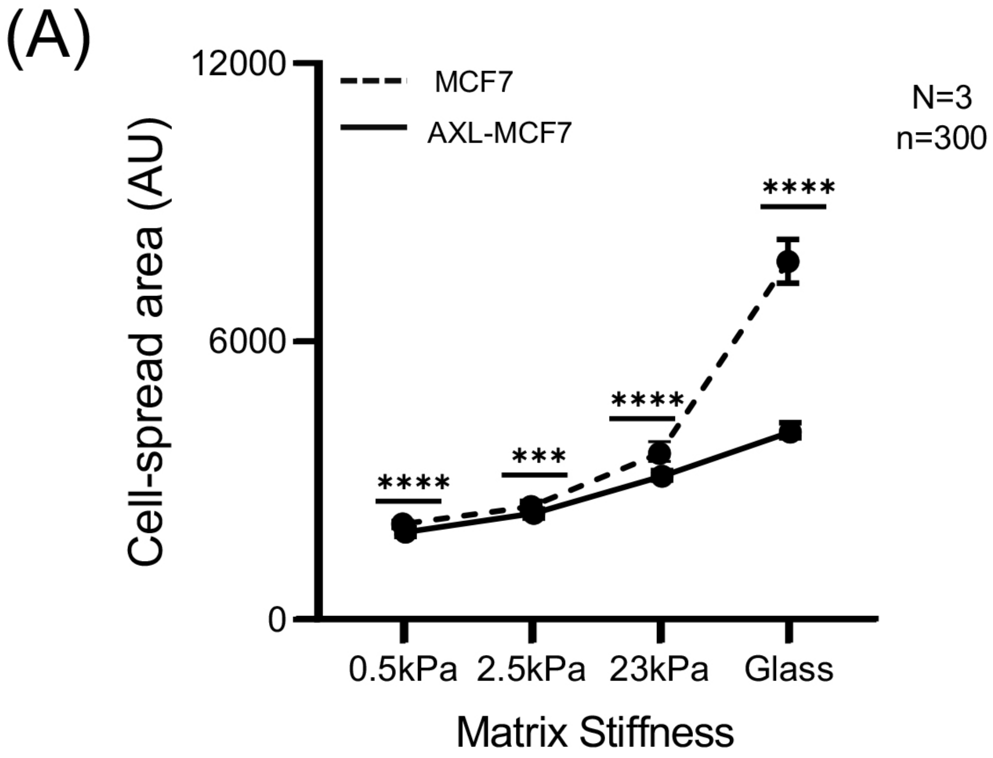

Fig. S4. (A) Cell spread area trends of MCF7 and AXL-MCF7 cells on gels of varying stiffness and glass. Graph represents trend for cell area for n=300 cells as median with 95% CI from N=3 experiments. Statistical analysis was done using Mann-Whitney U test. (*p≤0.05, **p ≤ 0.01, ***p ≤ 0.001, ****p ≤ 0.0001, ns=non-significant).

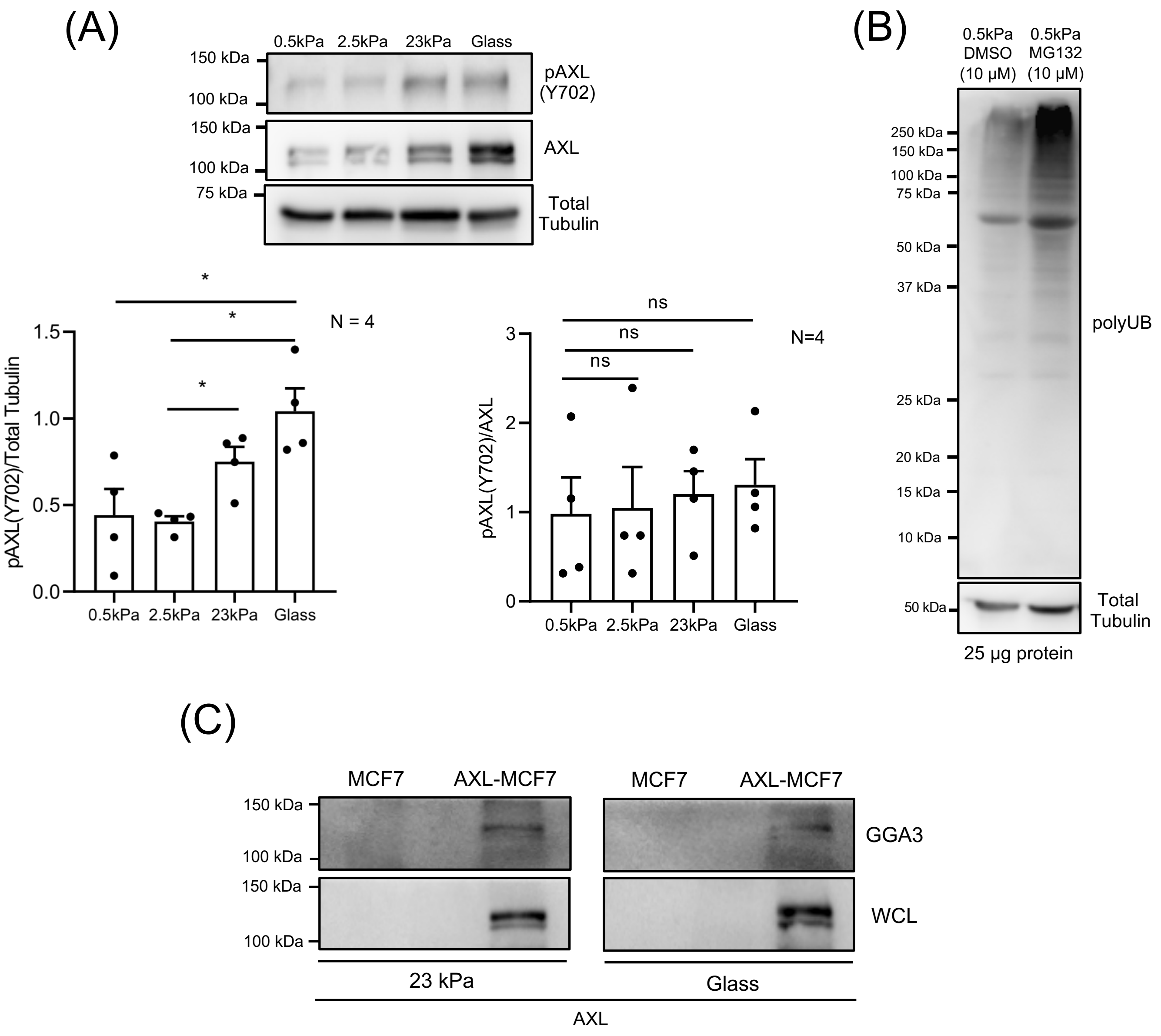

Fig. S5. Representative blots for **(A)** phospho-AXL (Y702), AXL and total-tubulin in MDAMB231 cells on gels of varying stiffness and glass. Graphs represent the ratio of densitometric band intensities (Left - pAXL/total tubulin) and (Right - pAXL/AXL) as mean ± SEM from N=4 experiments. The pAXL, AXL and tubulin blots were part of the same experiment presented in **Fig. 1E** and hence the loading control blot for total tubulin is the same. **(B)** Representative blots for Polyubiquitination (PolyUb) and total tubulin in 25 μg of cell lysates from DMSO and MG132 treated AXL-MCF7 cells on 0.5 kPa gel **(C)** Western blot detection of AXL in active Arf1 fraction (GGA3) and whole cell lysate (WCL) from MCF7 and AXL-MCF7 cells on 23kPa gel and glass. Statistical analysis was done using Mann-Whitney U test for western blot analysis. (*p≤0.05, **p ≤ 0.01, ***p ≤ 0.001, ****p ≤ 0.0001, ns=non-significant).

Journal of Cell Science • Supplementary information

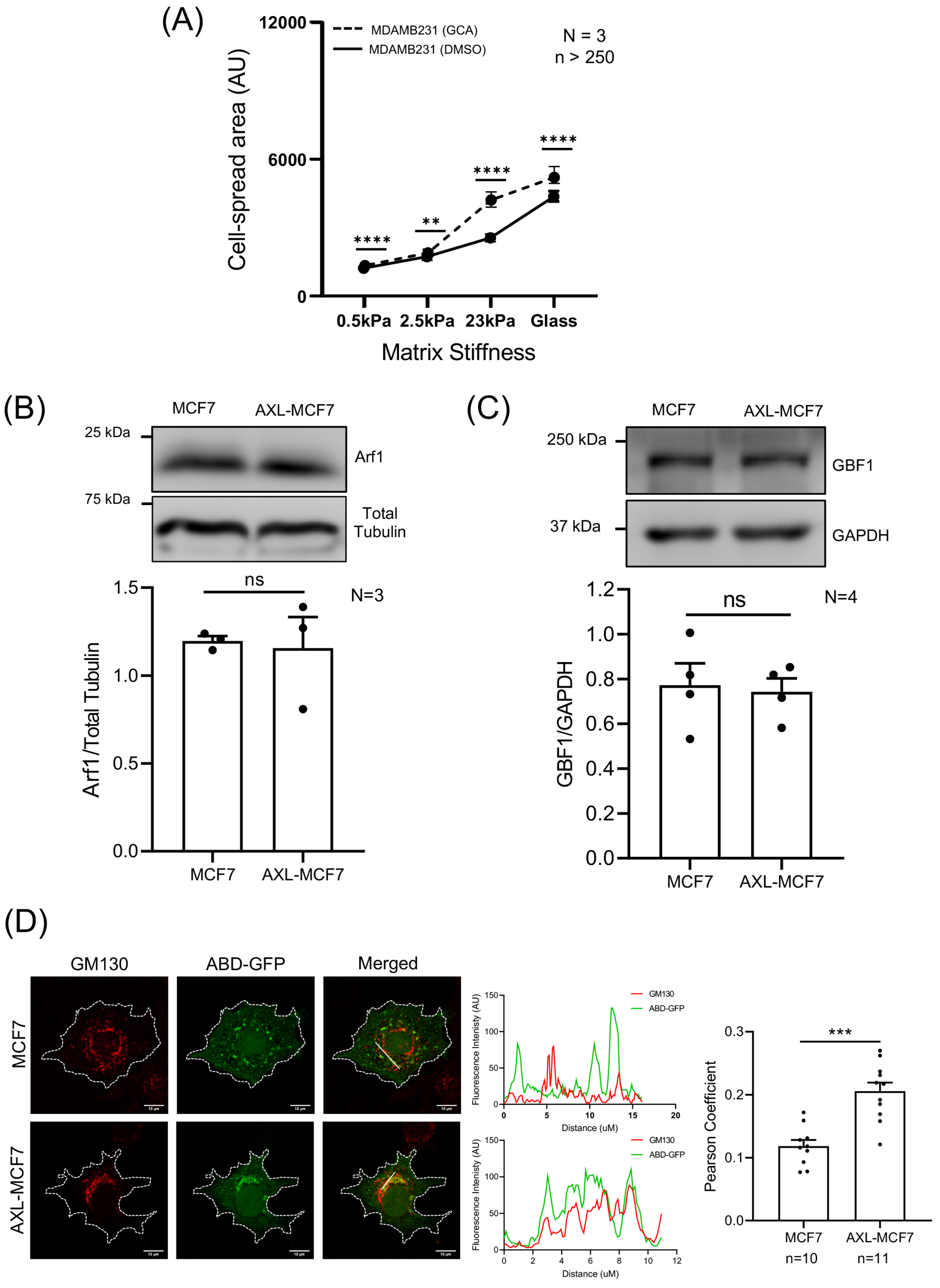

Journal of Cell Science • Supplementary information

(E)

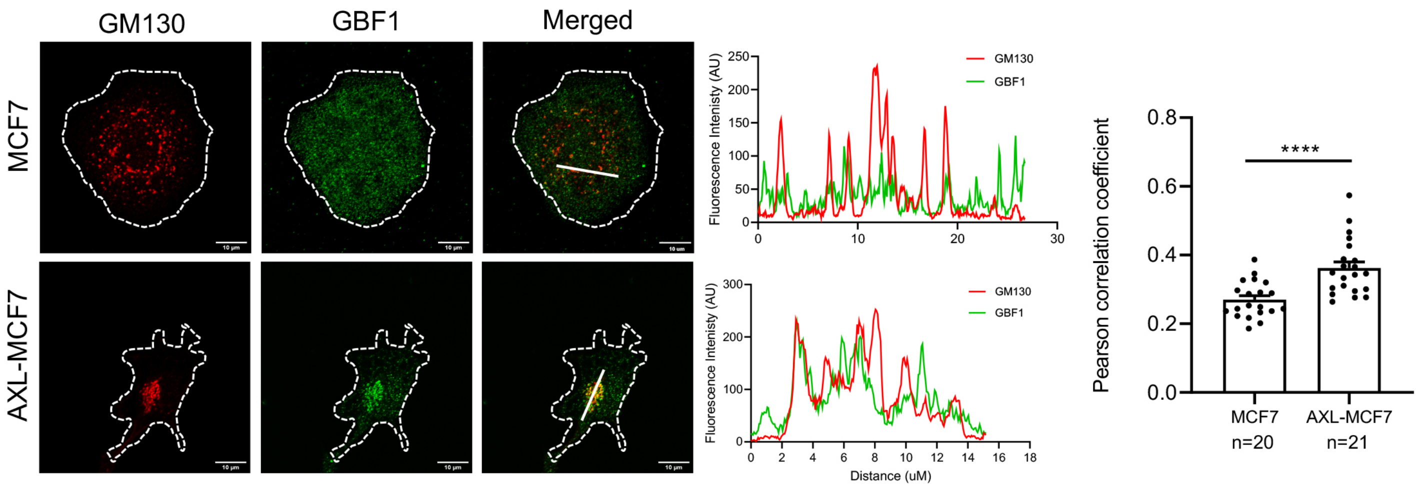

Fig. S6. (A) Cell spread area trends of DMSO and GCA treated MDAMB231 cells on gels of varying stiffness and glass. Graph represents trend for cell area for n=300 cells as median and 95%CI from N=3 experiments. **(B, C)** Representative blots of **(B)** Arf1 and total-tubulin and **(C)** GBF1 and GAPDH levels for MCF7 vs. AXL-MCF7 cells. Graphs represent the ratio of densitometric band intensities (Arf1/total tubulin and GBF1/GAPDH) as mean ± SEM from N=3 and N=4 experiments.**(D, E)** Cross-section images and intensity line plots for MCF7 and AXL-MCF7 cells expressing **(D)** ABD-GFP (green) and **(E)** immunostained for GBF1 (green), both with immunostaining for GM130 (red). Graphs represent the Pearson's correlation coefficients for colocalization of **(D)** ABD-GFP (green) and GM130 (red) as mean ± SEM for n=10 and 11 cells and **(E)** GBF1 (green) and GM130 (red) as mean ± SEM for n=20 and 21 cells for N=2 experiments. Statistical analysis was done using Mann-Whitney U test for cell area, colocalization and western blot analysis. (*p≤0.05, **p ≤ 0.01, ***p ≤ 0.001, ****p ≤ 0.0001, ns=non-significant)

Journal of Cell Science • Supplementary information

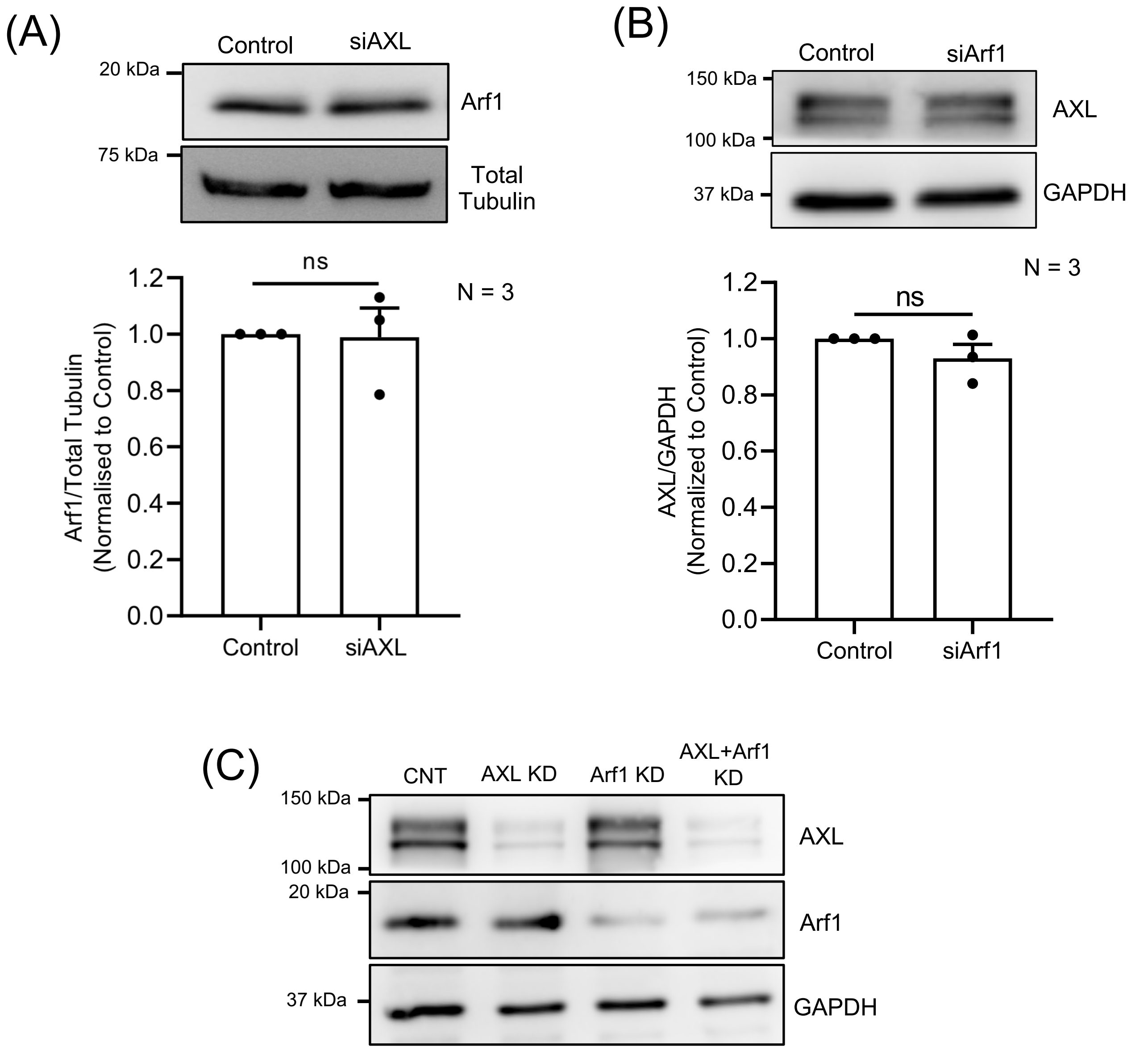

Fig. S7. (A) Representative blots for Arf1 and total-tubulin in control and AXL knockdown (siAXL) MDAMB231 cells. The Arf1 and total tubulin blots were part of the same experiment presented in Fig. S2K and hence the loading control blot for total tubulin is the same. **(B)** Representative blots for AXL and GAPDH in control and Arf1 knockdown (siArf1) MDAMB231 cells. Graph represents the ratio of densitometric band intensities (normalised to control) as mean ± SEM from N=3 experiments for both Fig. S7A & B. **(C)** Representative blot for AXL, Arf1 and GAPDH in control, AXL KD, Arf1 KD, and AXL+Arf1 KD MDAMB231 cells. Statistical analysis was done using Single sample Wilcoxon t test for western blots analysis normalised (with respect to control). (*p≤0.05, **p ≤ 0.01, ***p ≤ 0.001, ****p ≤ 0.0001, ns=non-significant)

Journal of Cell Science • Supplementary information

Figure 1

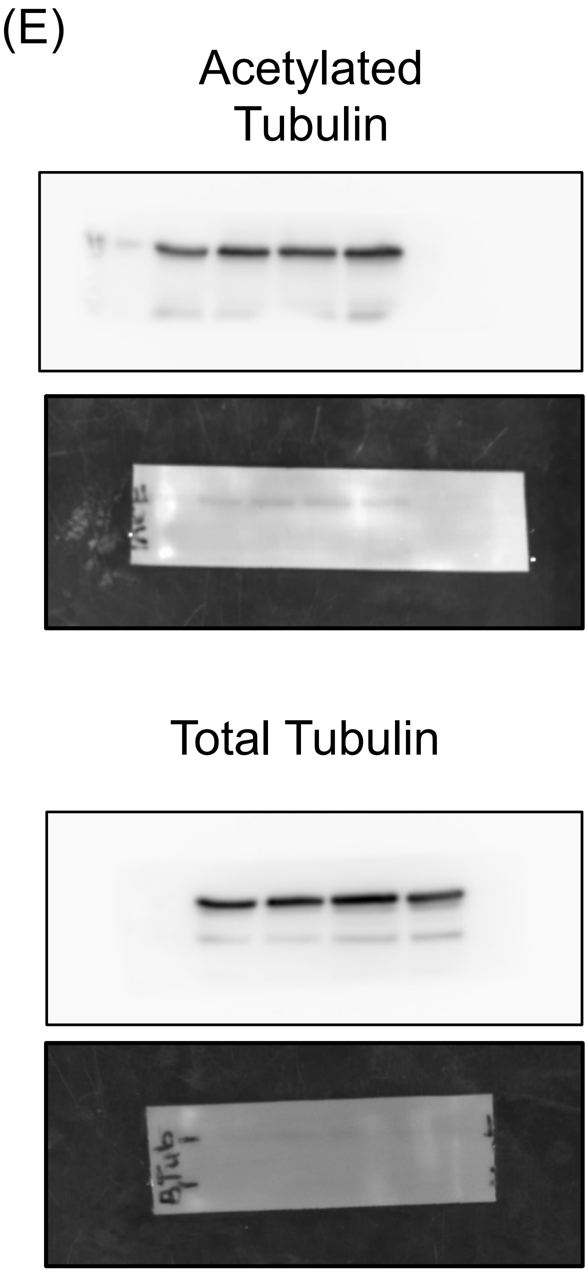

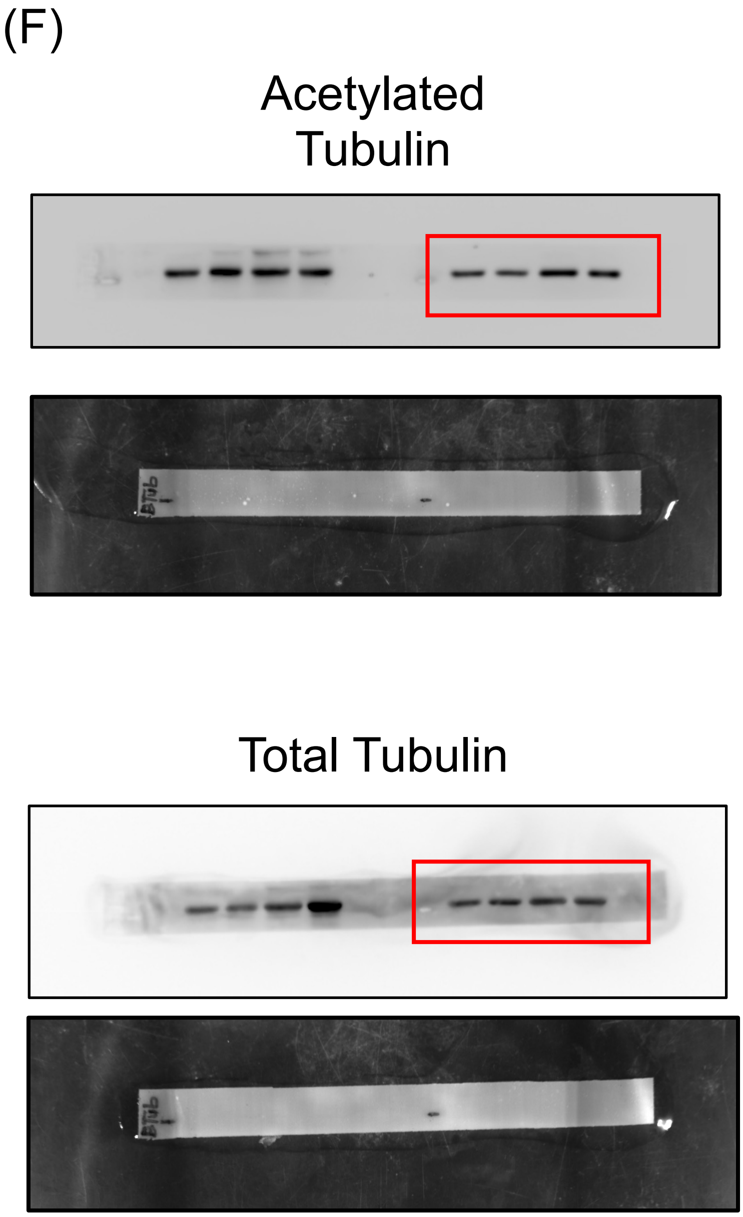

Journal of Cell Science • Supplementary information

J. Cell Sci.: doi:10.1242/jcs.263956: Supplementary information

Fig S1

(C)

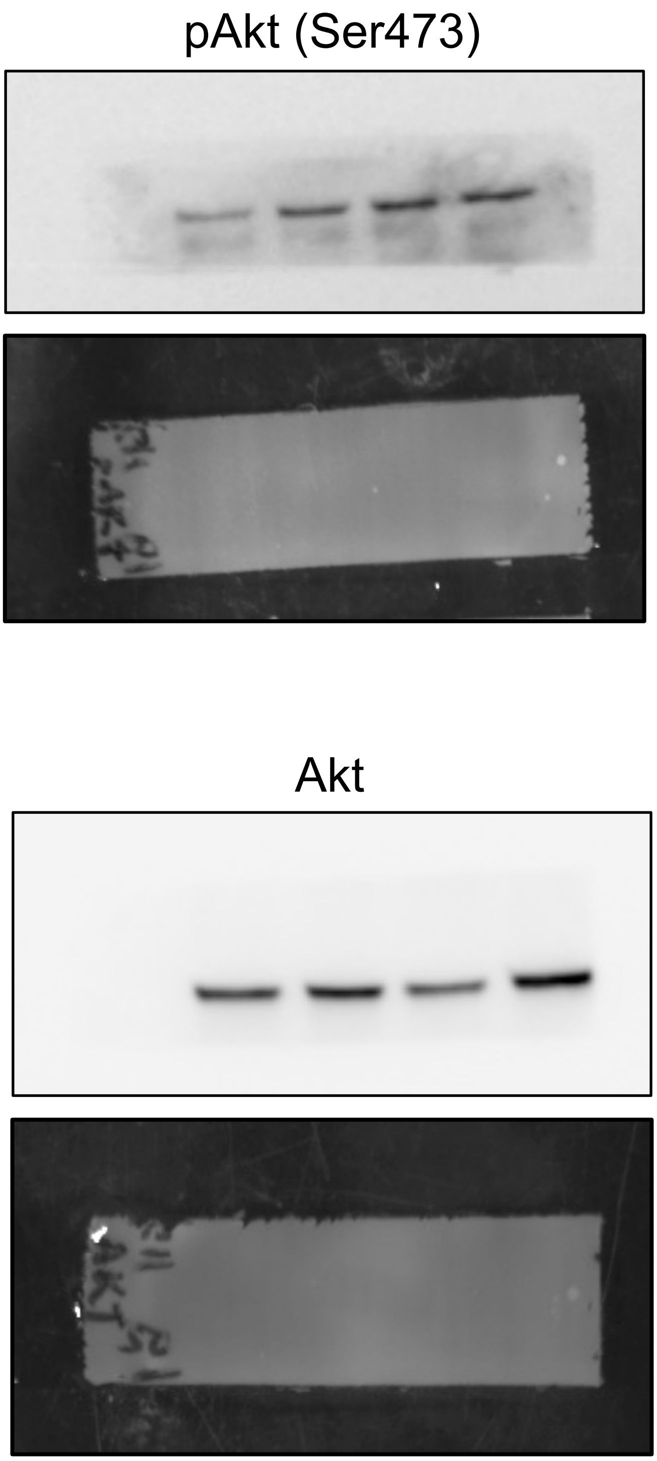

Journal of Cell Science • Supplementary information

Fig S2

(A)

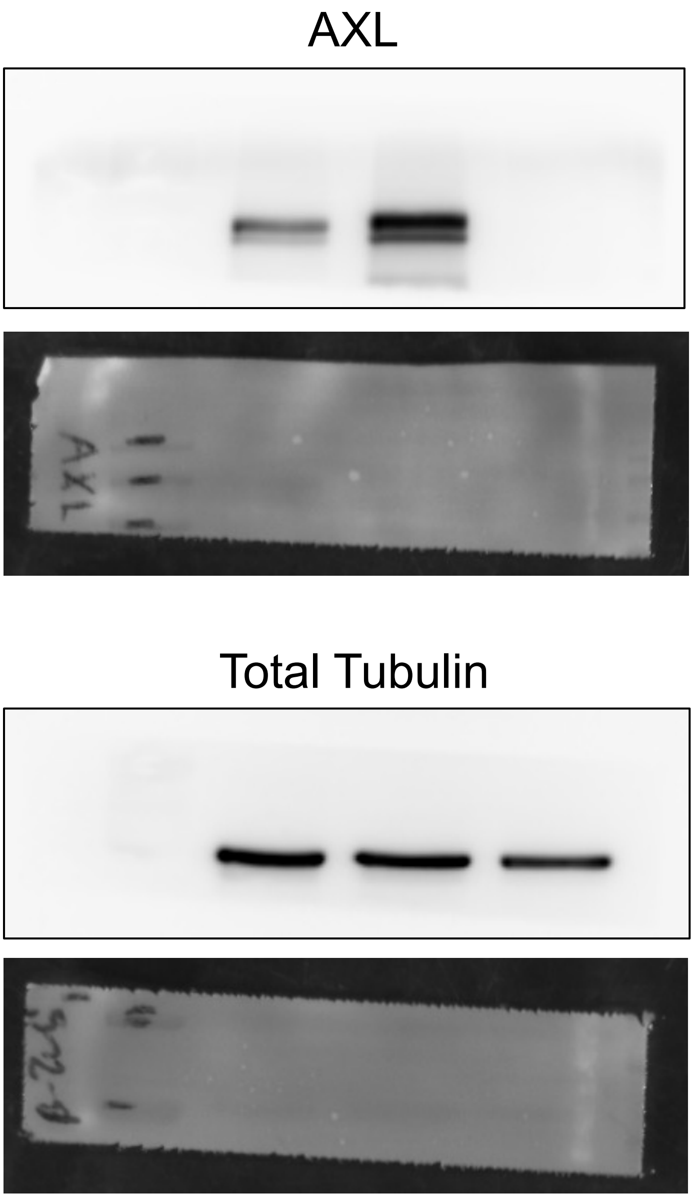

(D)

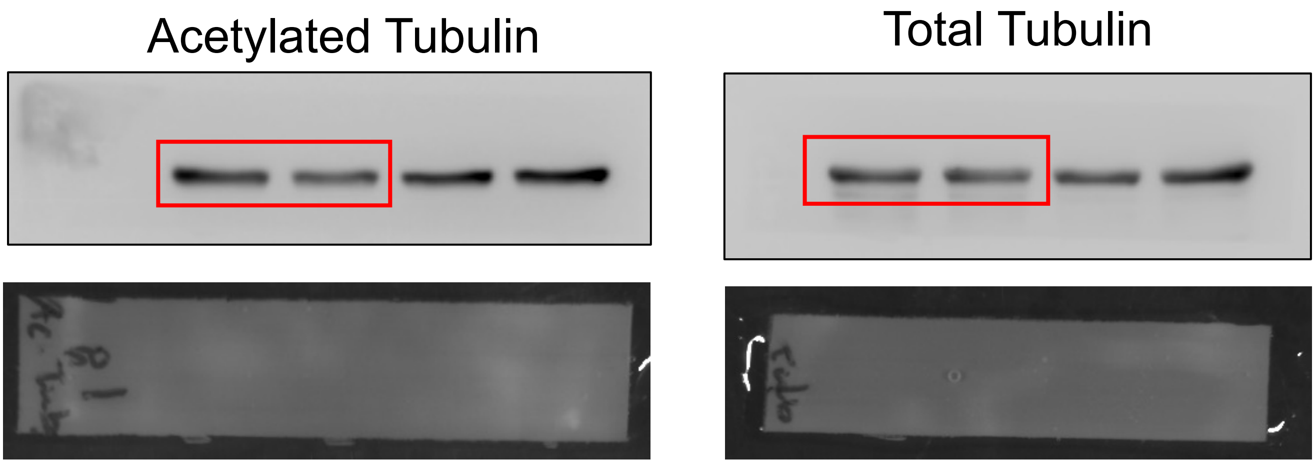

Journal of Cell Science • Supplementary information

Fig S2

(G)

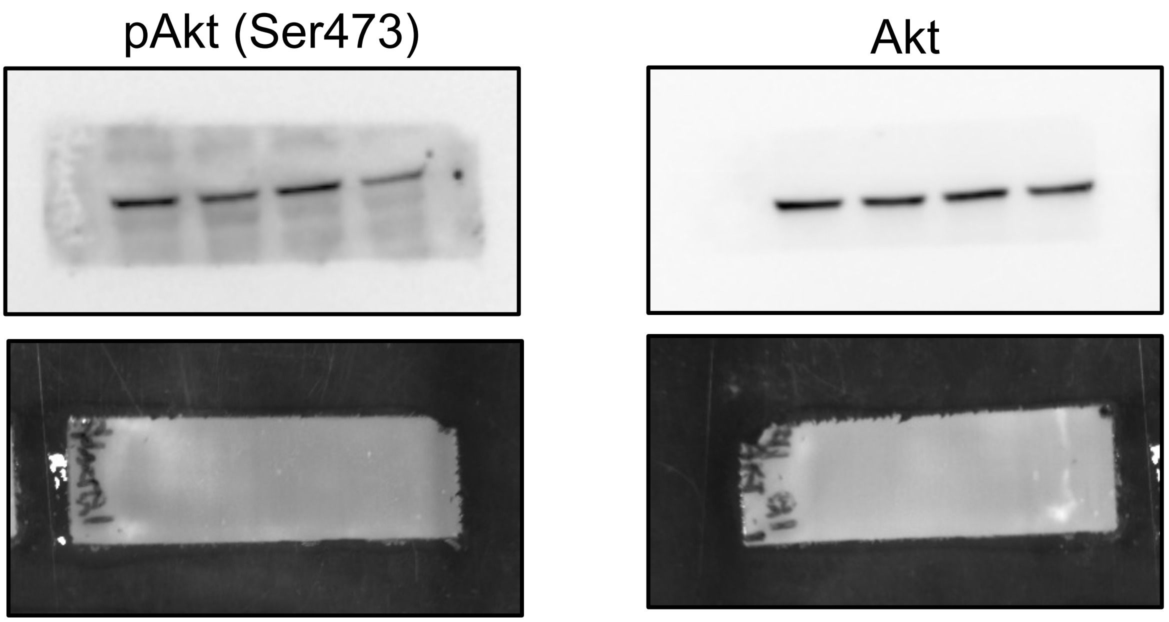

(H)

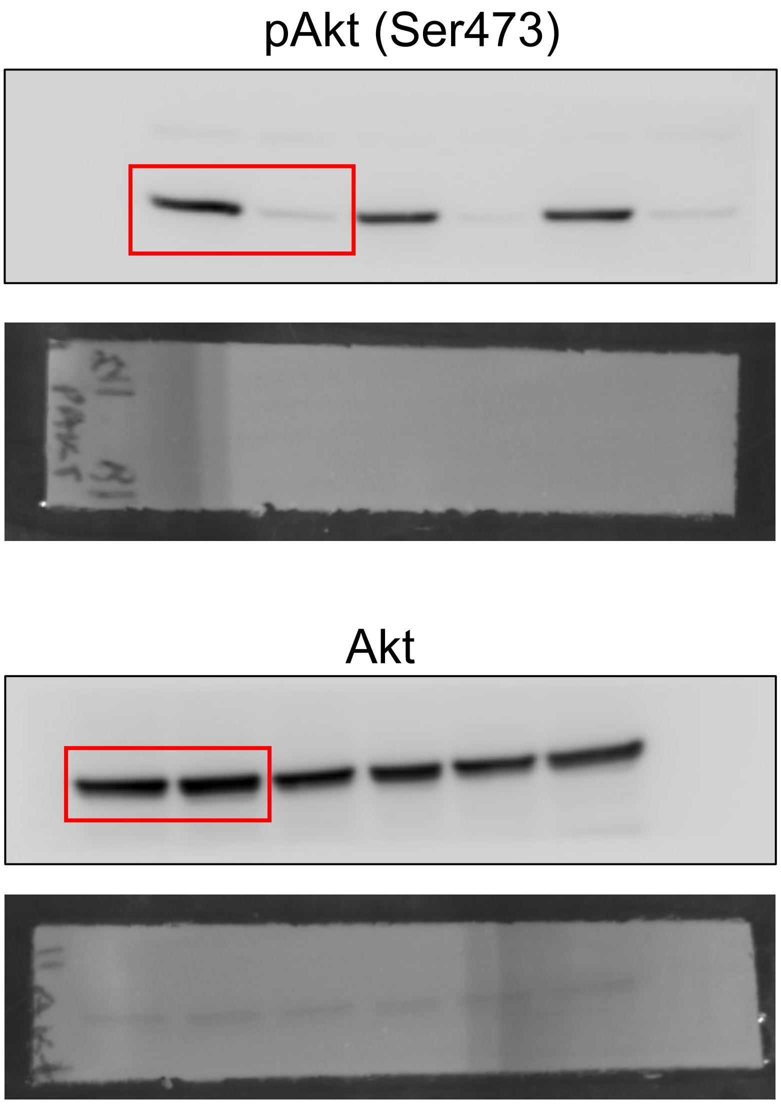

Journal of Cell Science • Supplementary information

Fig S2

(I)

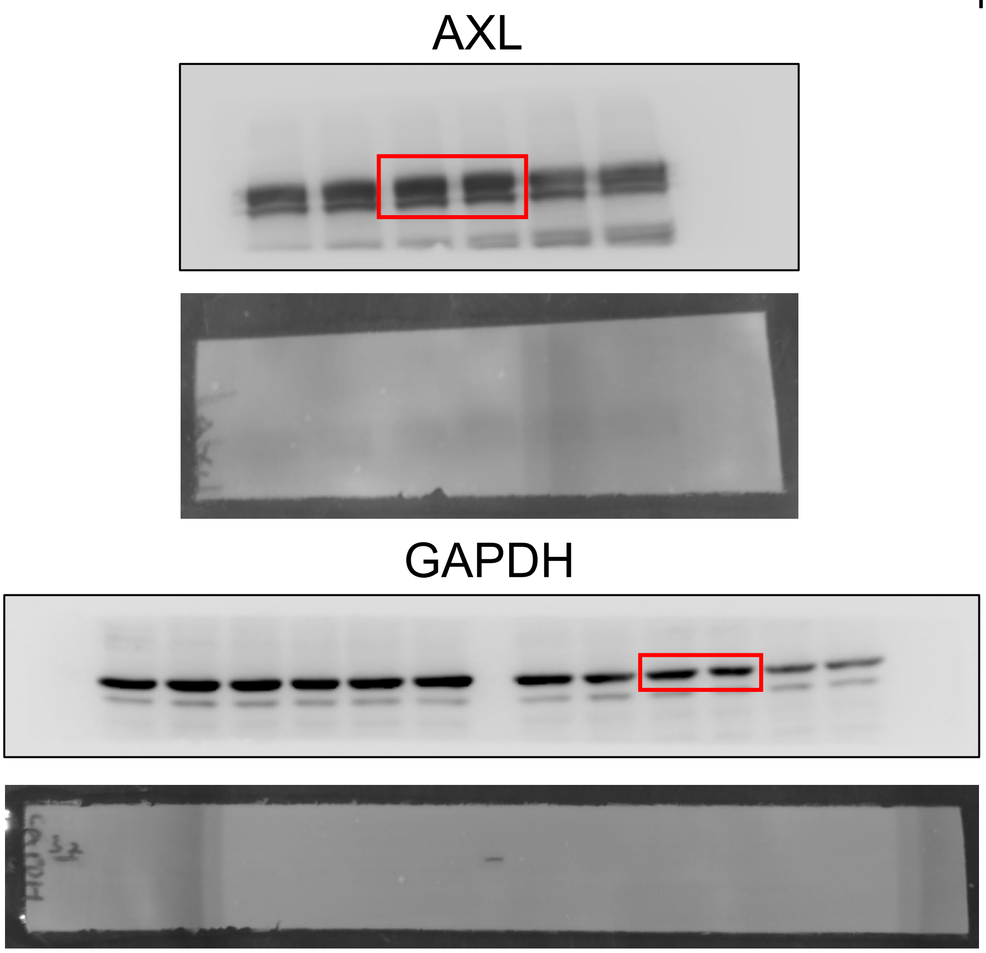

(K)

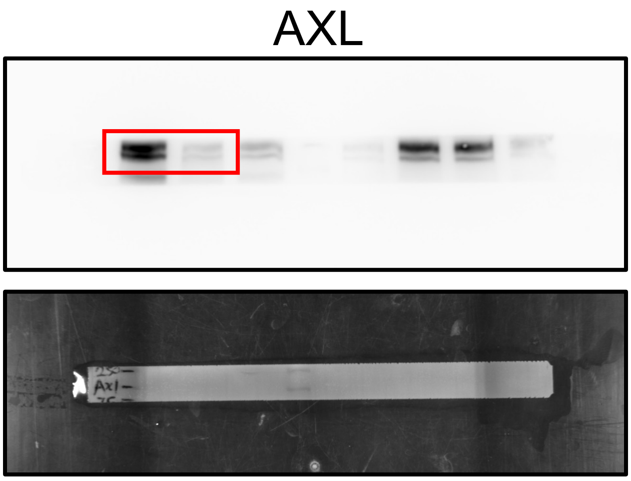

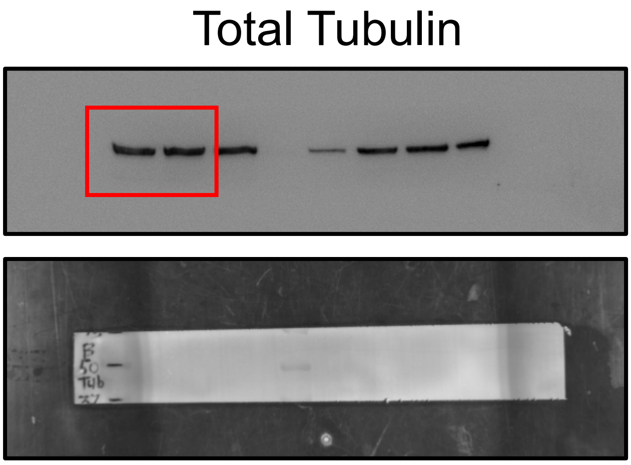

Journal of Cell Science • Supplementary information

Figure 3

(B)

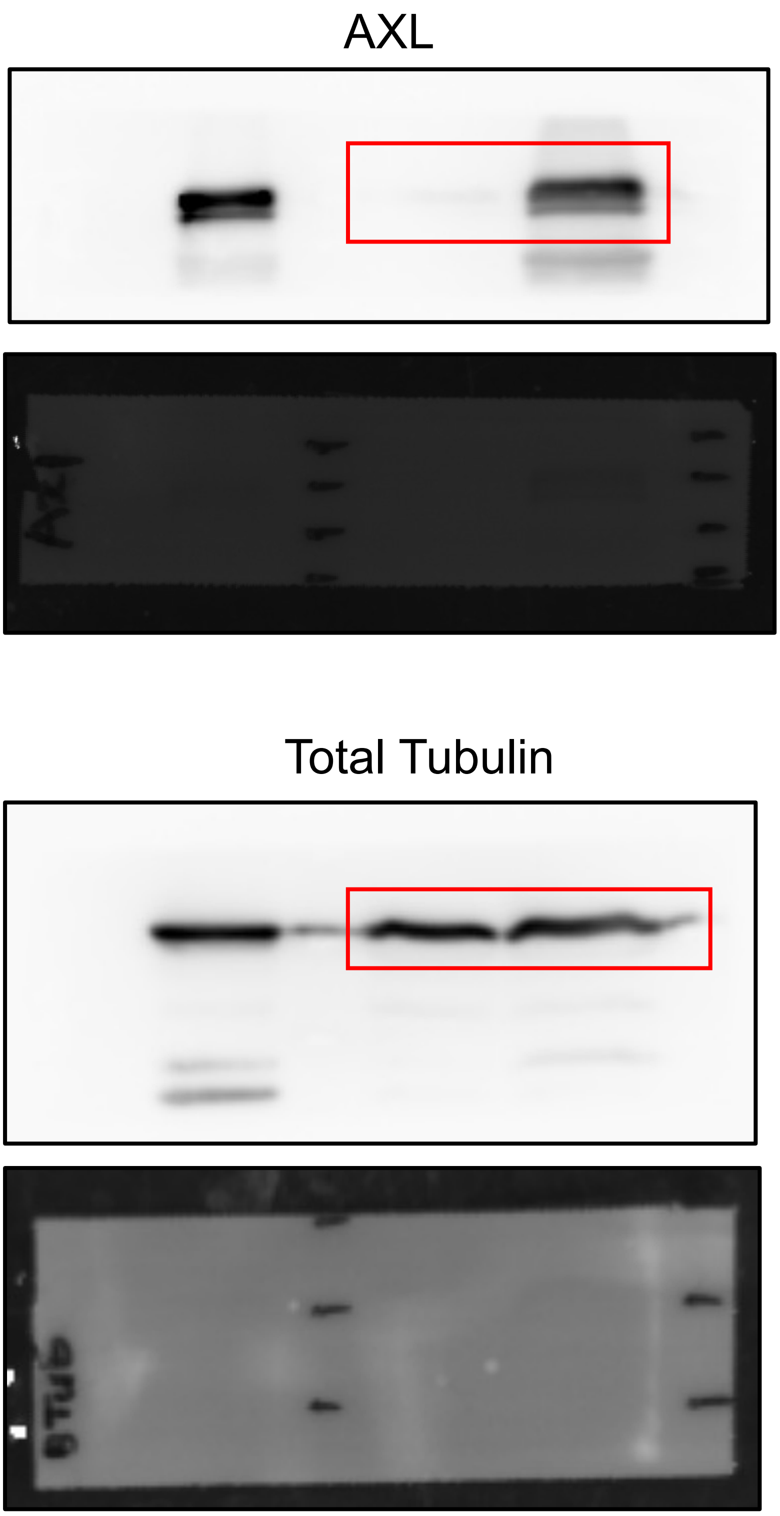

Figure 5

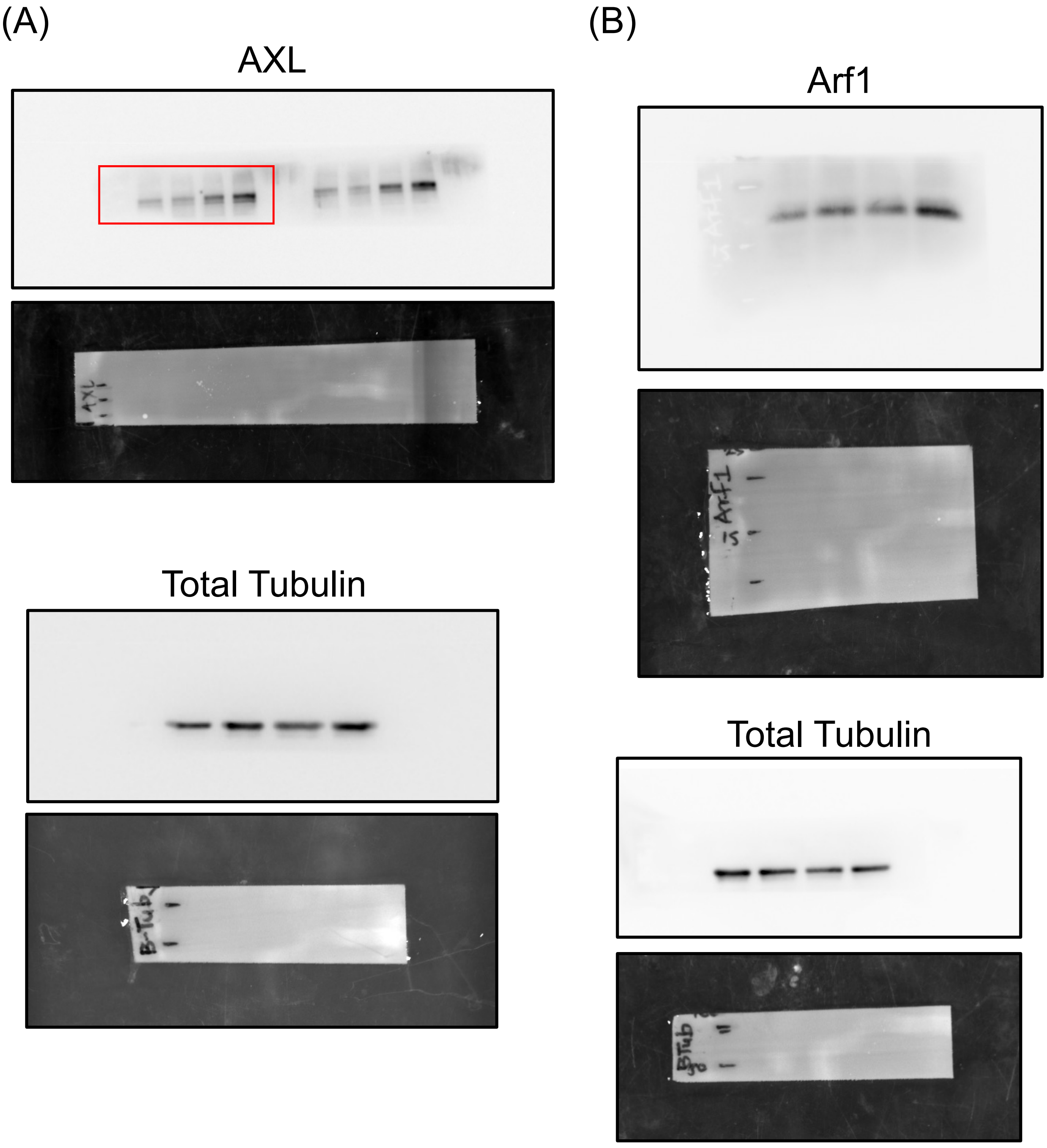

Journal of Cell Science • Supplementary information

Figure 5

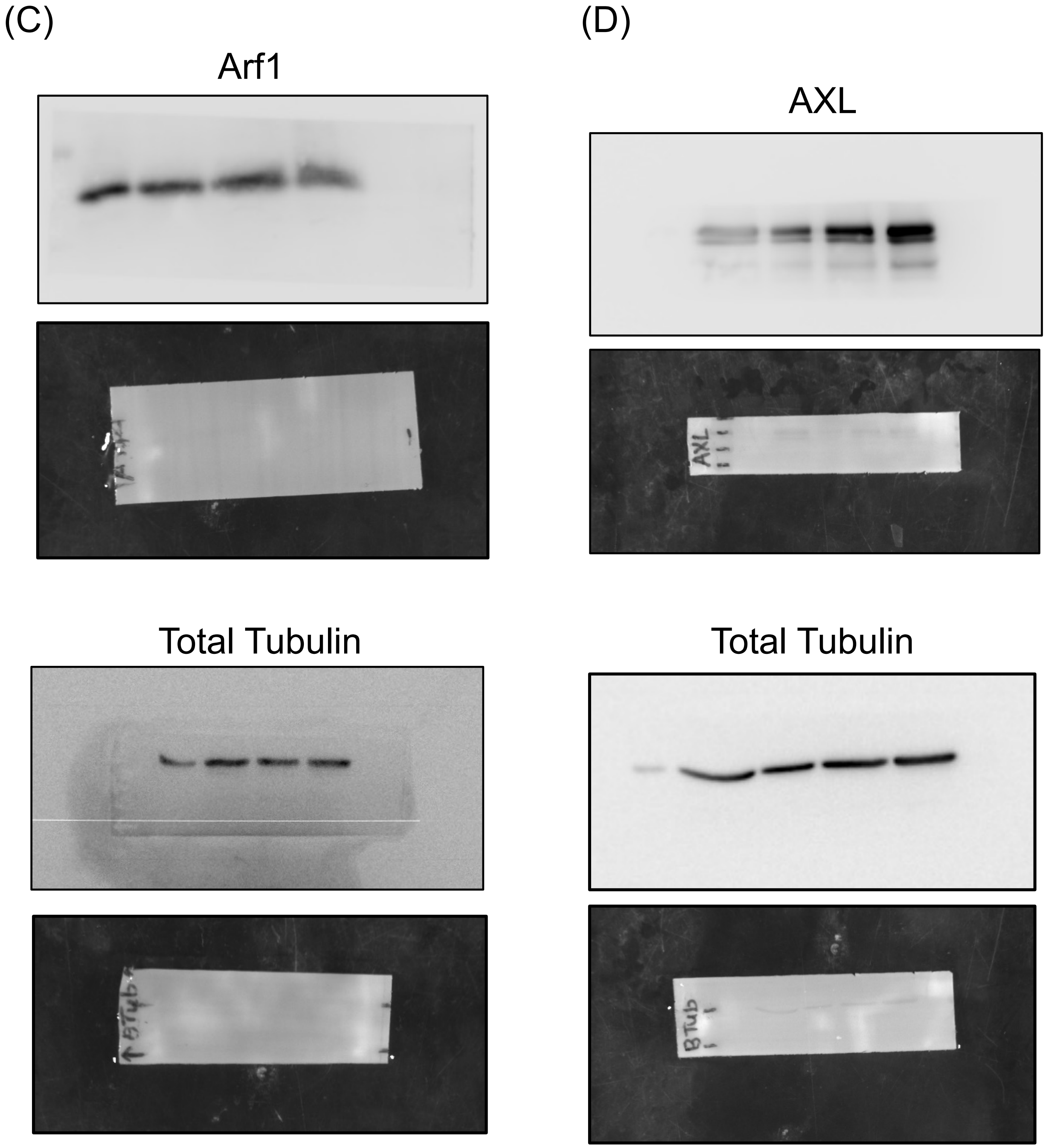

Journal of Cell Science • Supplementary information

Figure 5

(E)

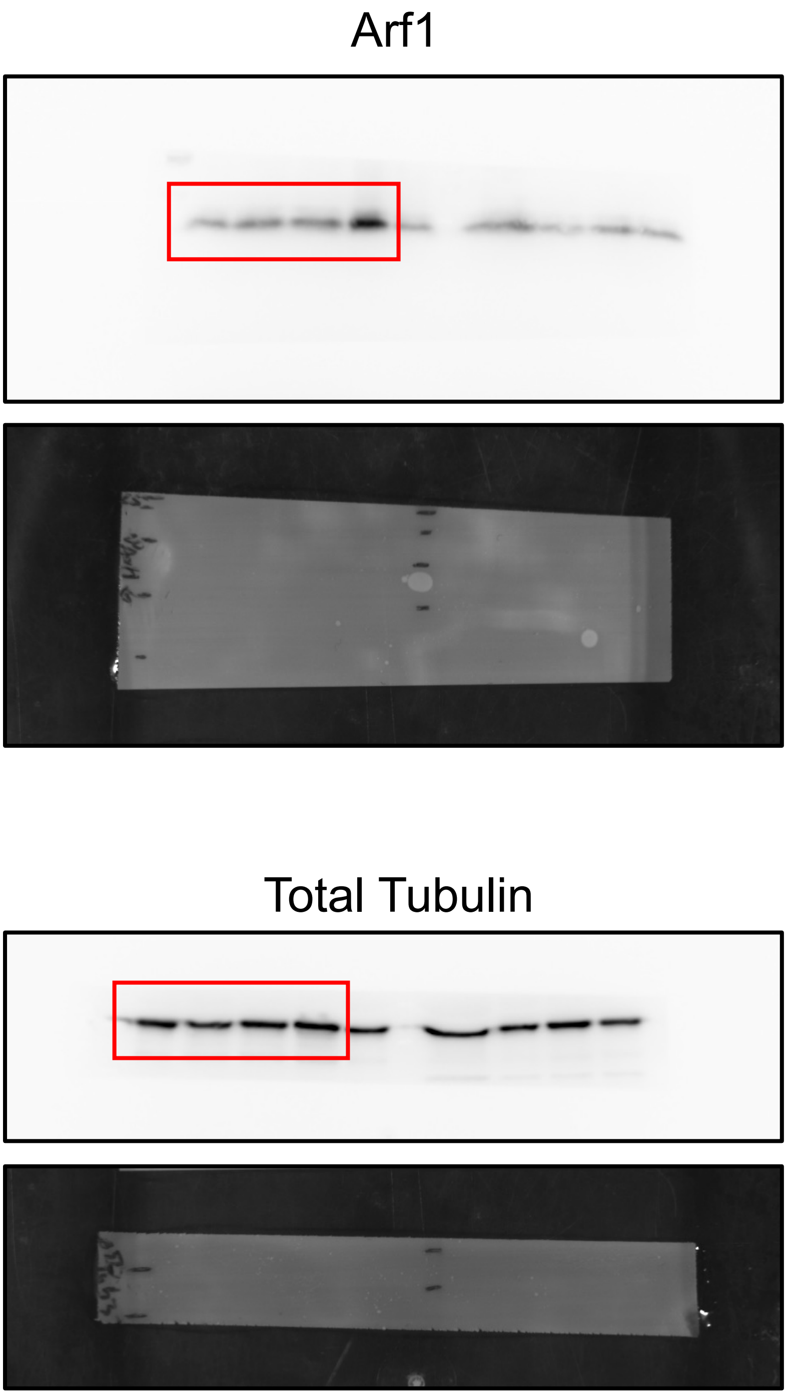

Journal of Cell Science • Supplementary information

Figure 5

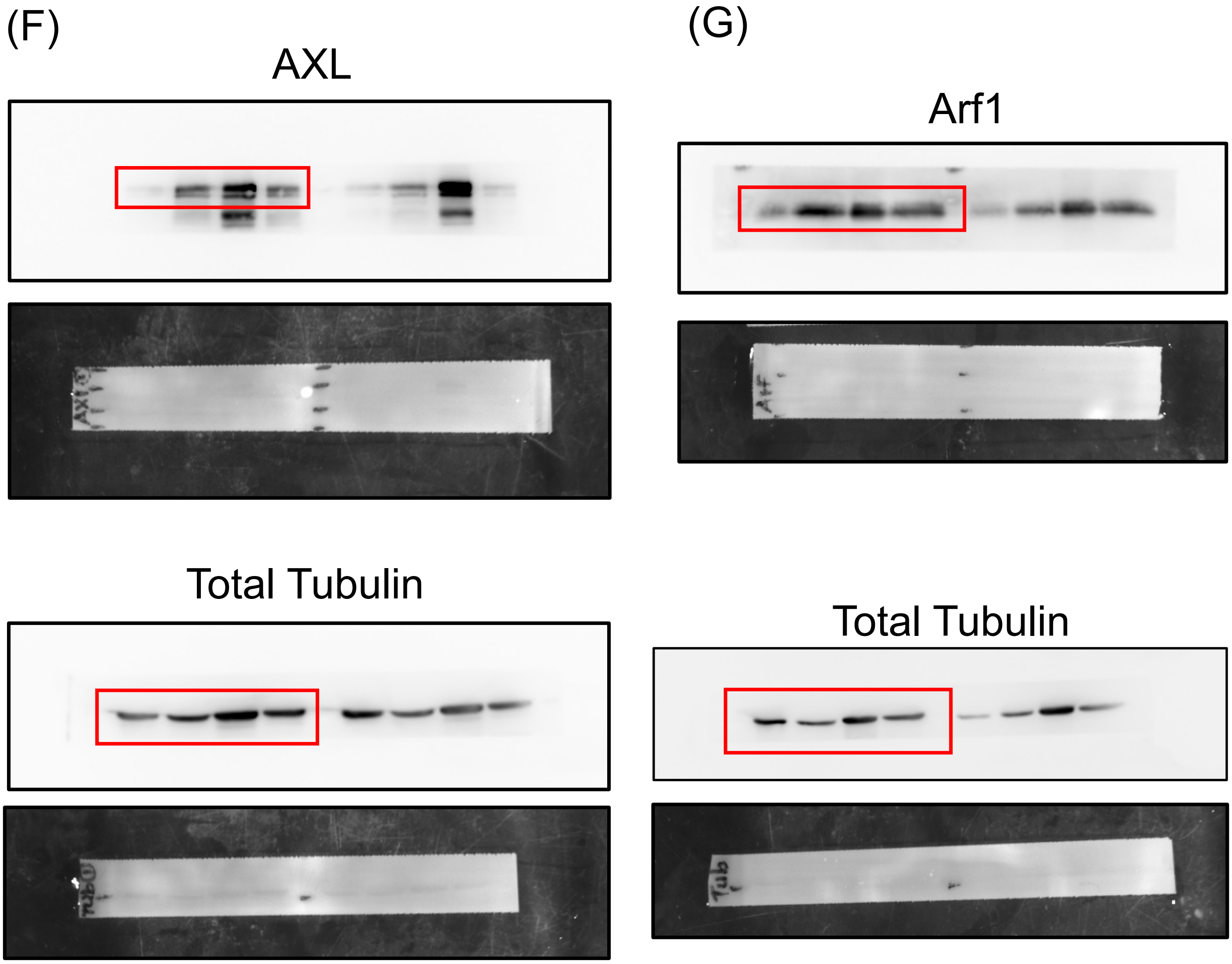

Journal of Cell Science • Supplementary information

Figure 5

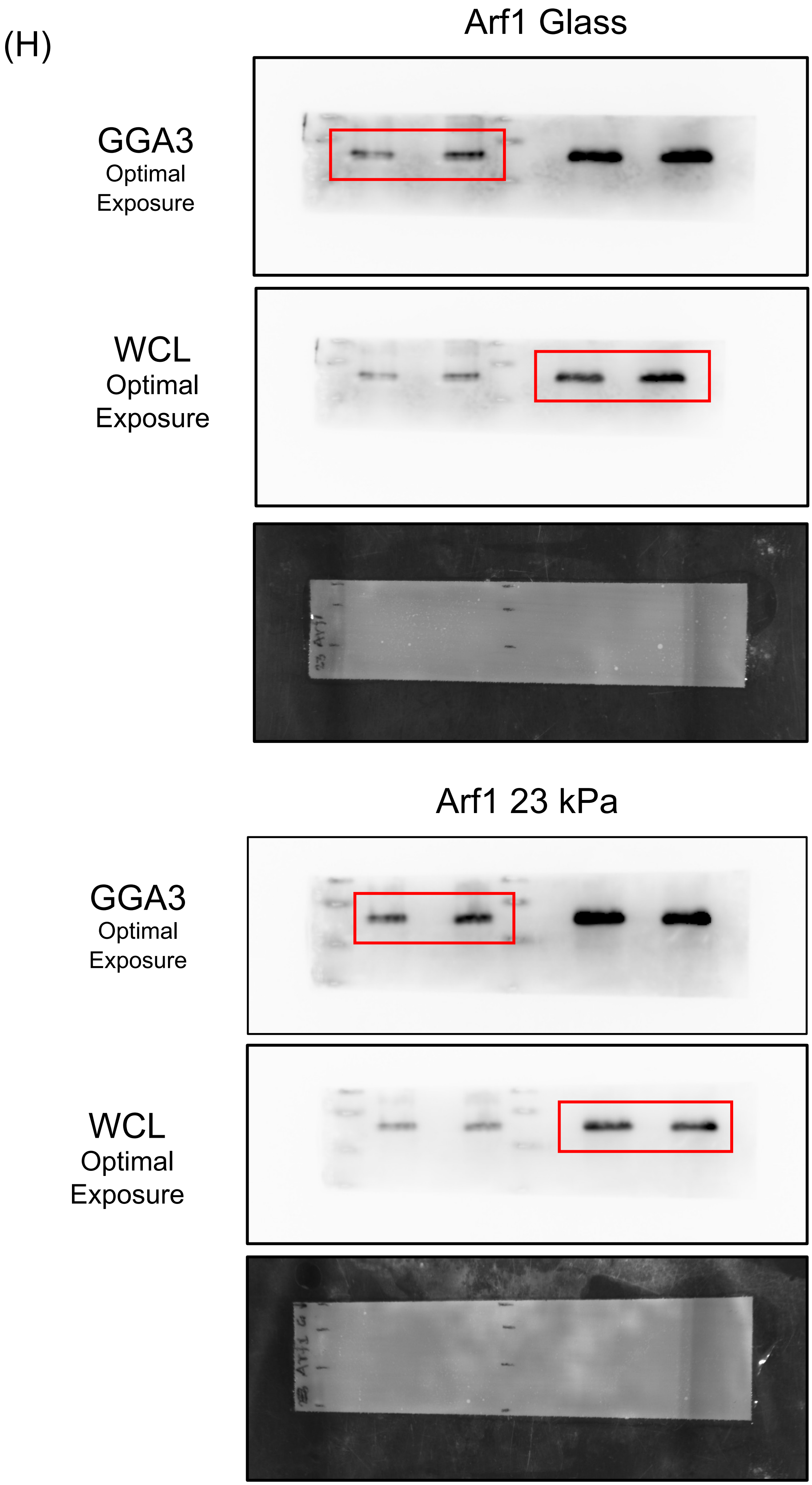

Journal of Cell Science • Supplementary information

Fig S5

(A)

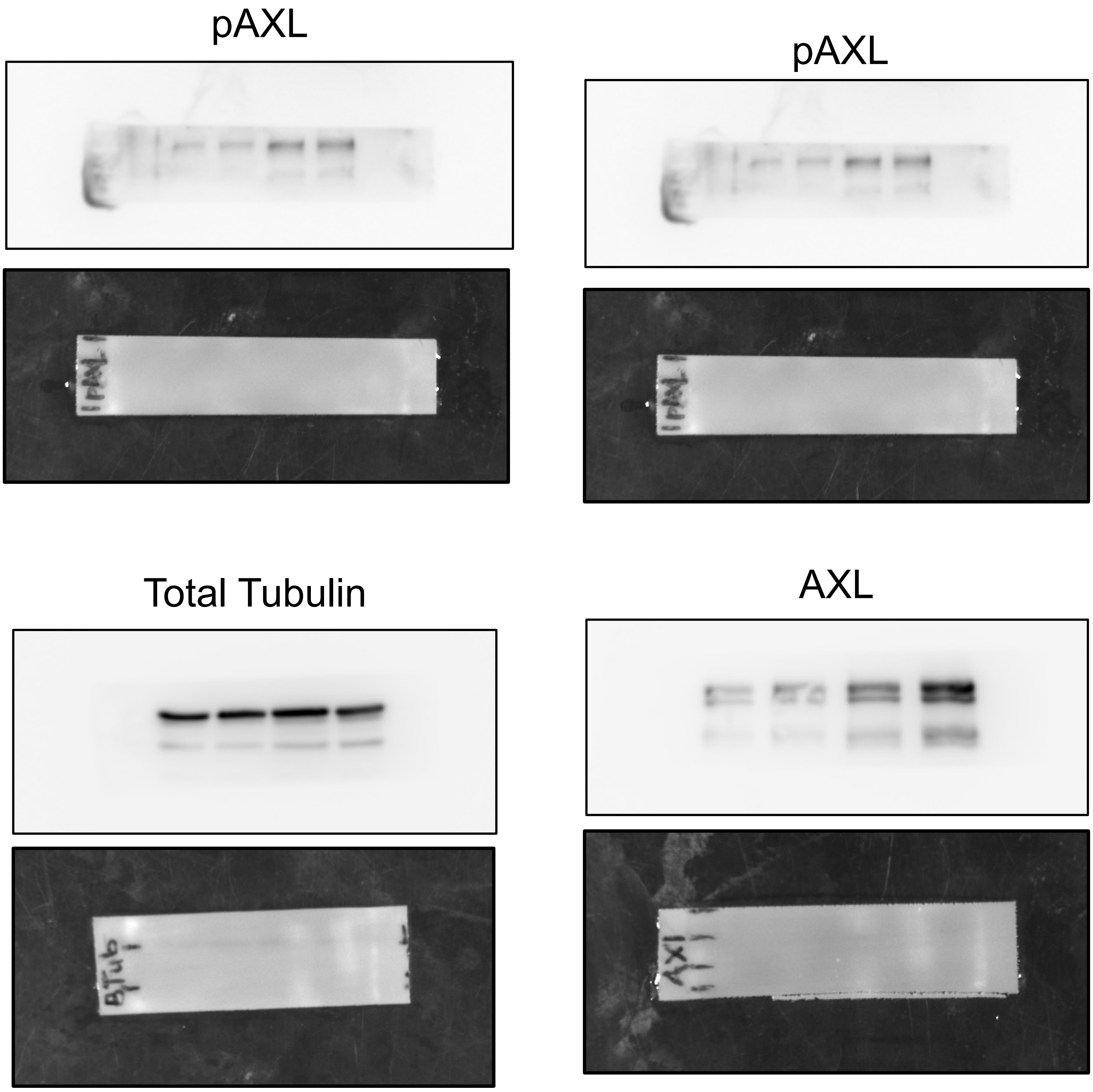

Journal of Cell Science • Supplementary information

Fig S5

(B)

polyUB

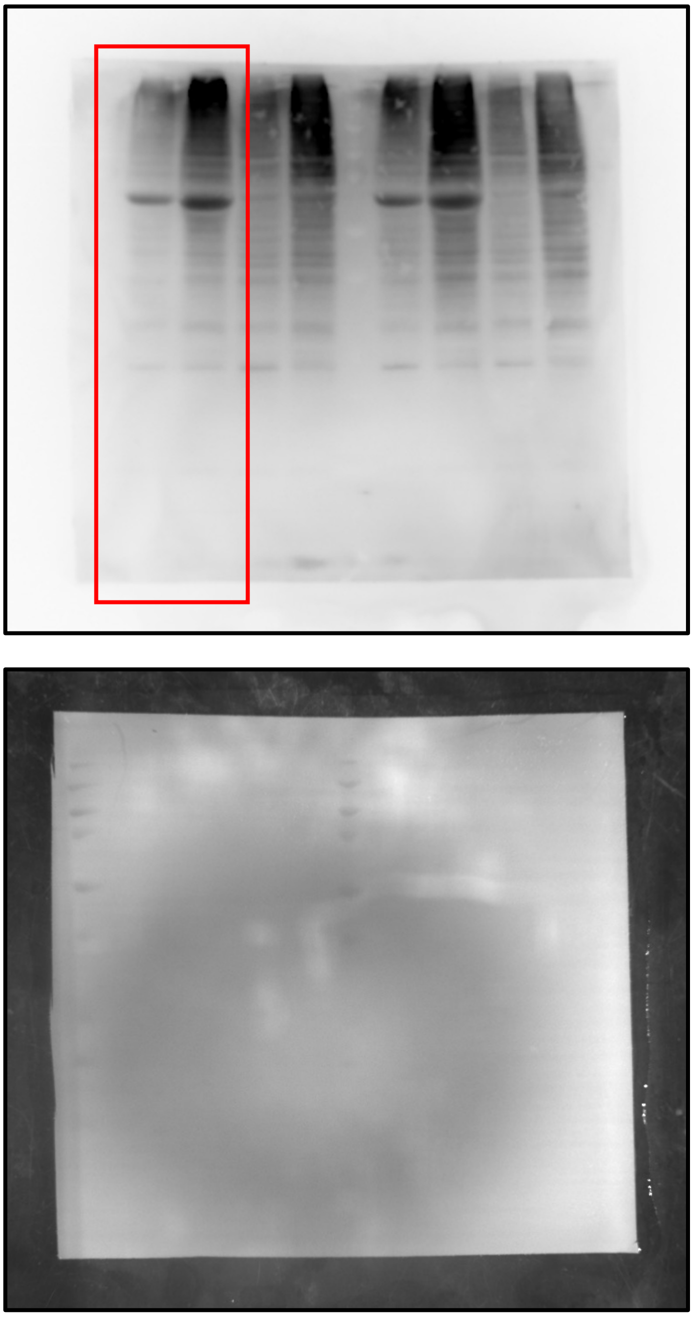

Total Tubulin

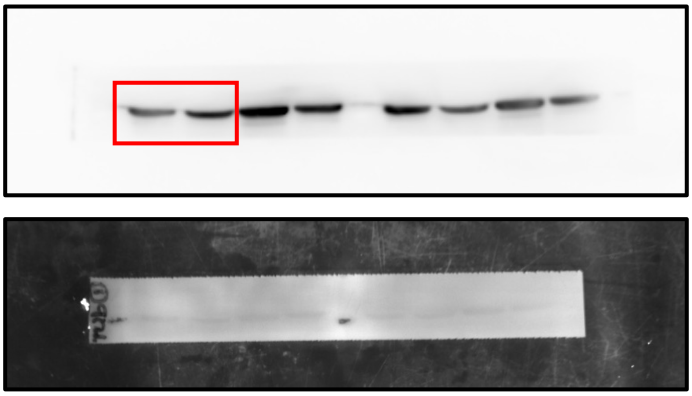

Journal of Cell Science • Supplementary information

Fig S5

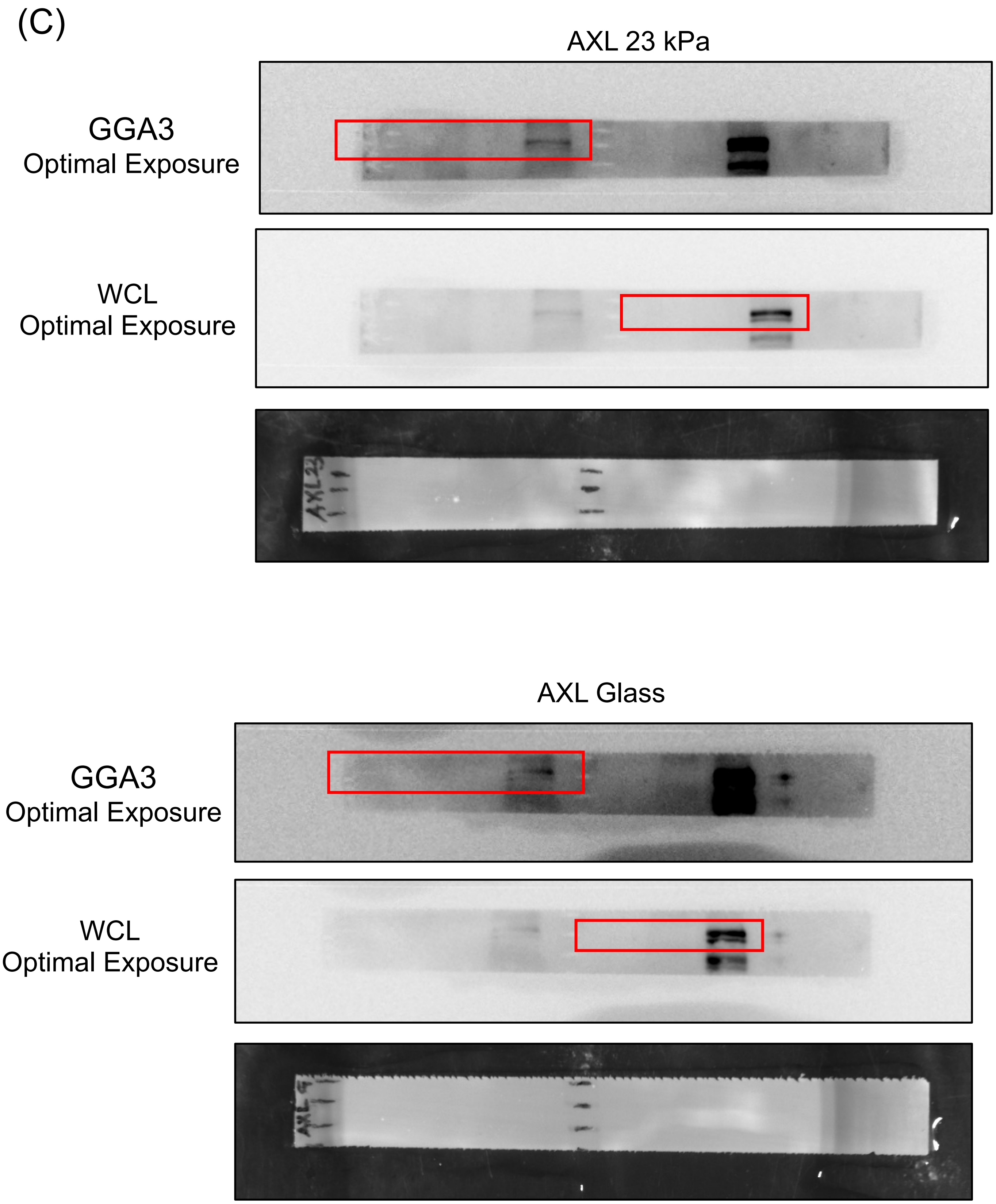
(C)
AXL 23 kPa
GGA3
Optimal Exposure
WCL
Optimal Exposure
AXL Glass
GGA3
Optimal Exposure
WCL
Optimal Exposure

Figure 6

(D)

Acetylated Tubulin

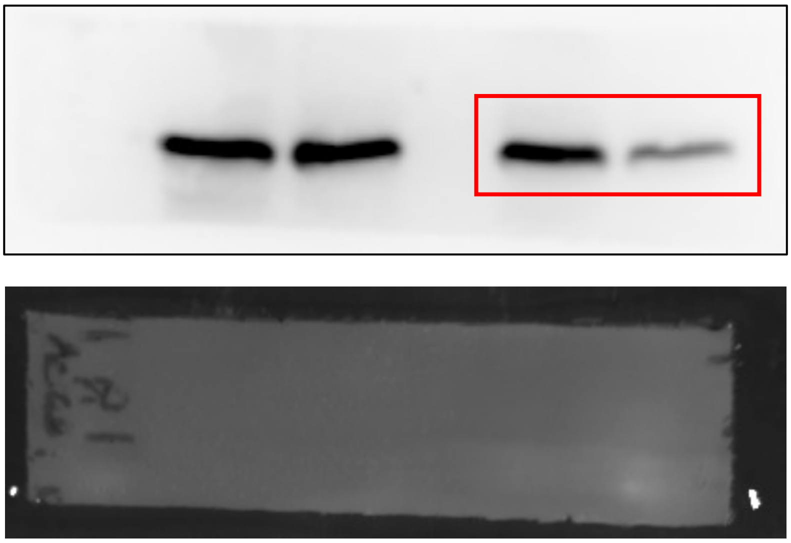

Total Tubulin

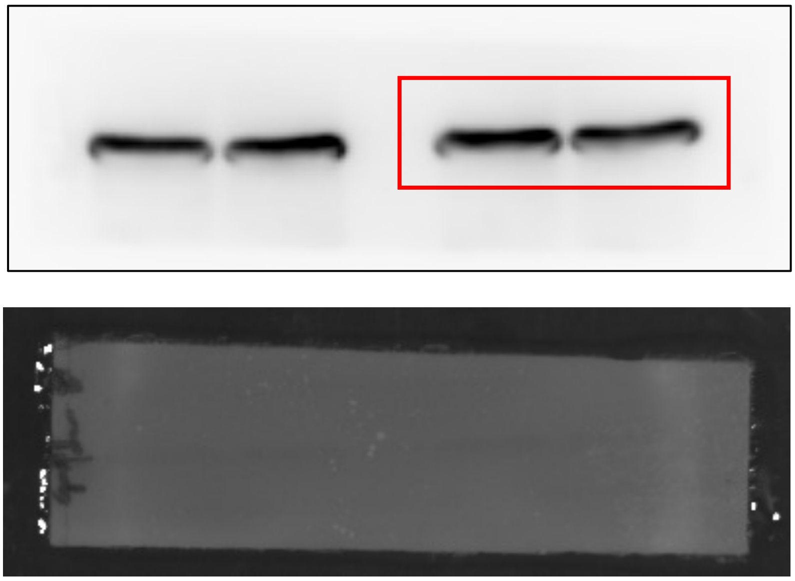

Journal of Cell Science • Supplementary information

Fig S6

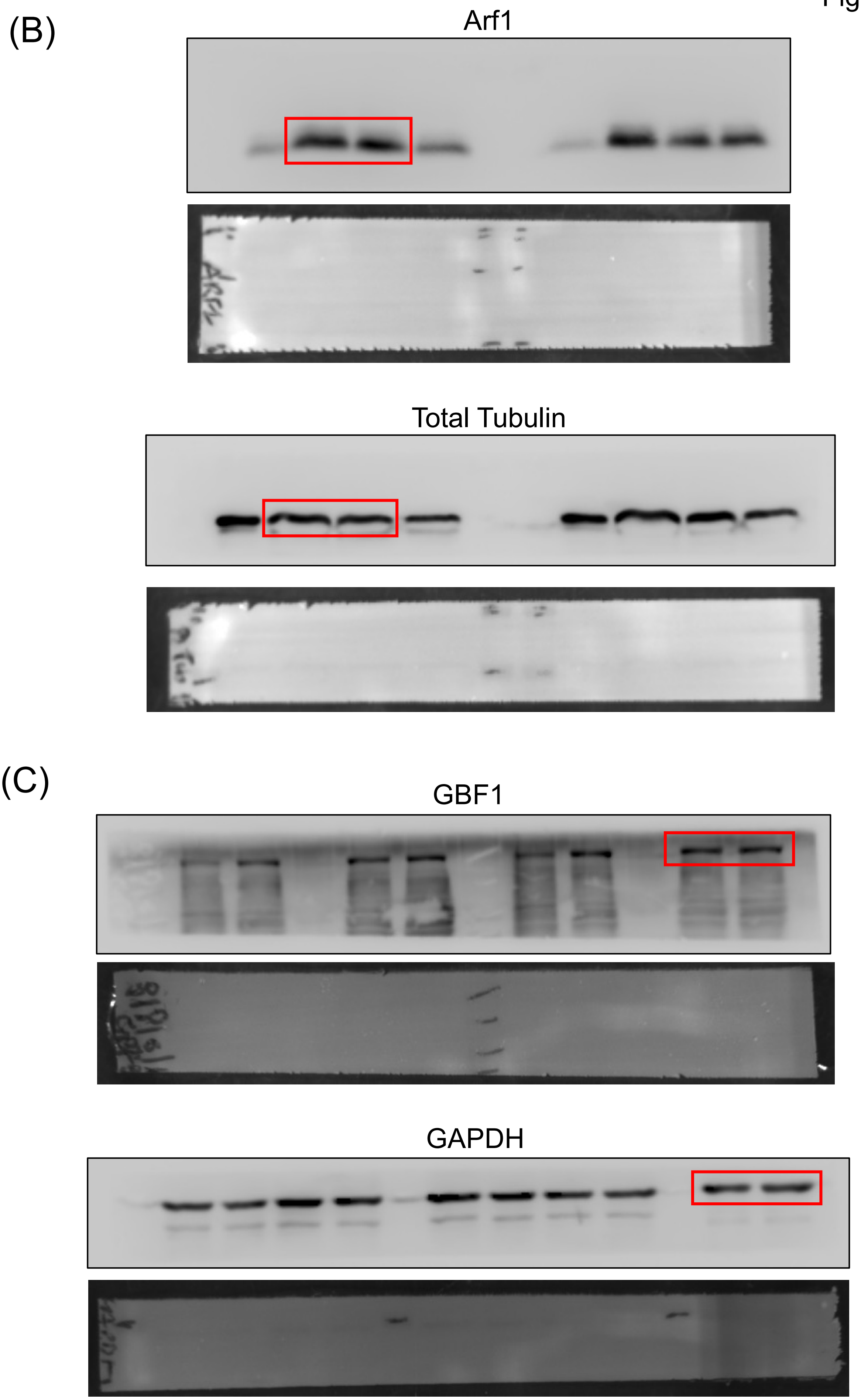

Journal of Cell Science • Supplementary information

Figure 7

(B)

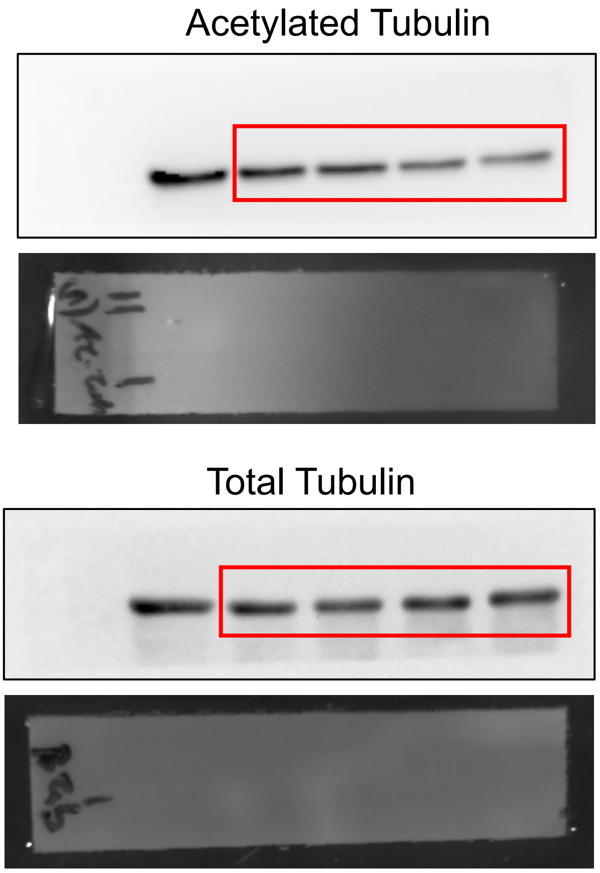

(D)

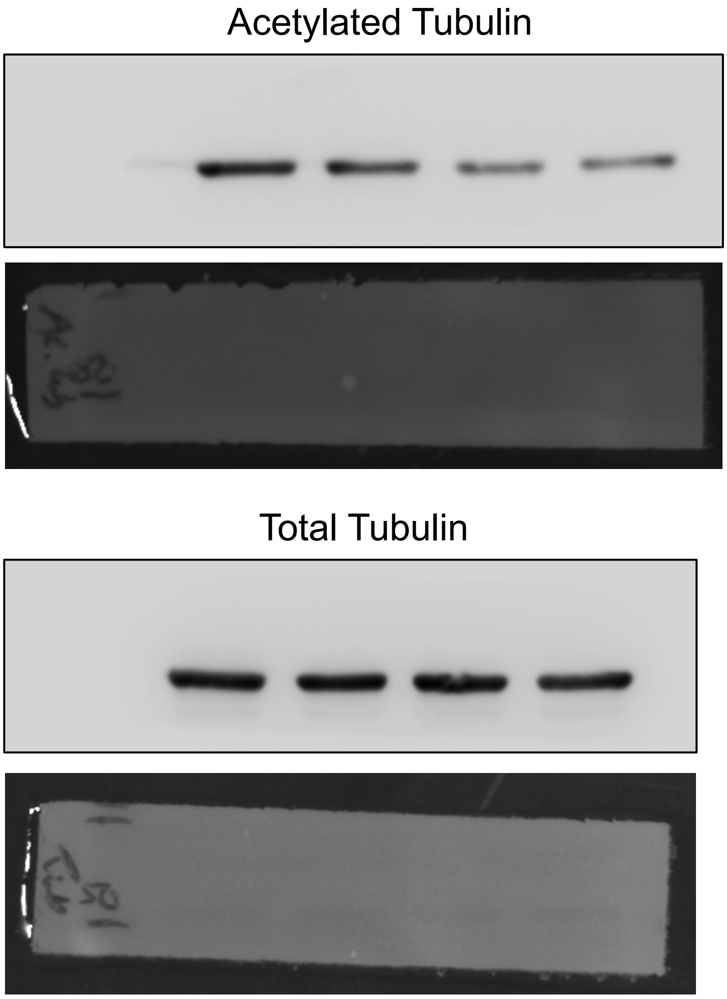

Journal of Cell Science • Supplementary information

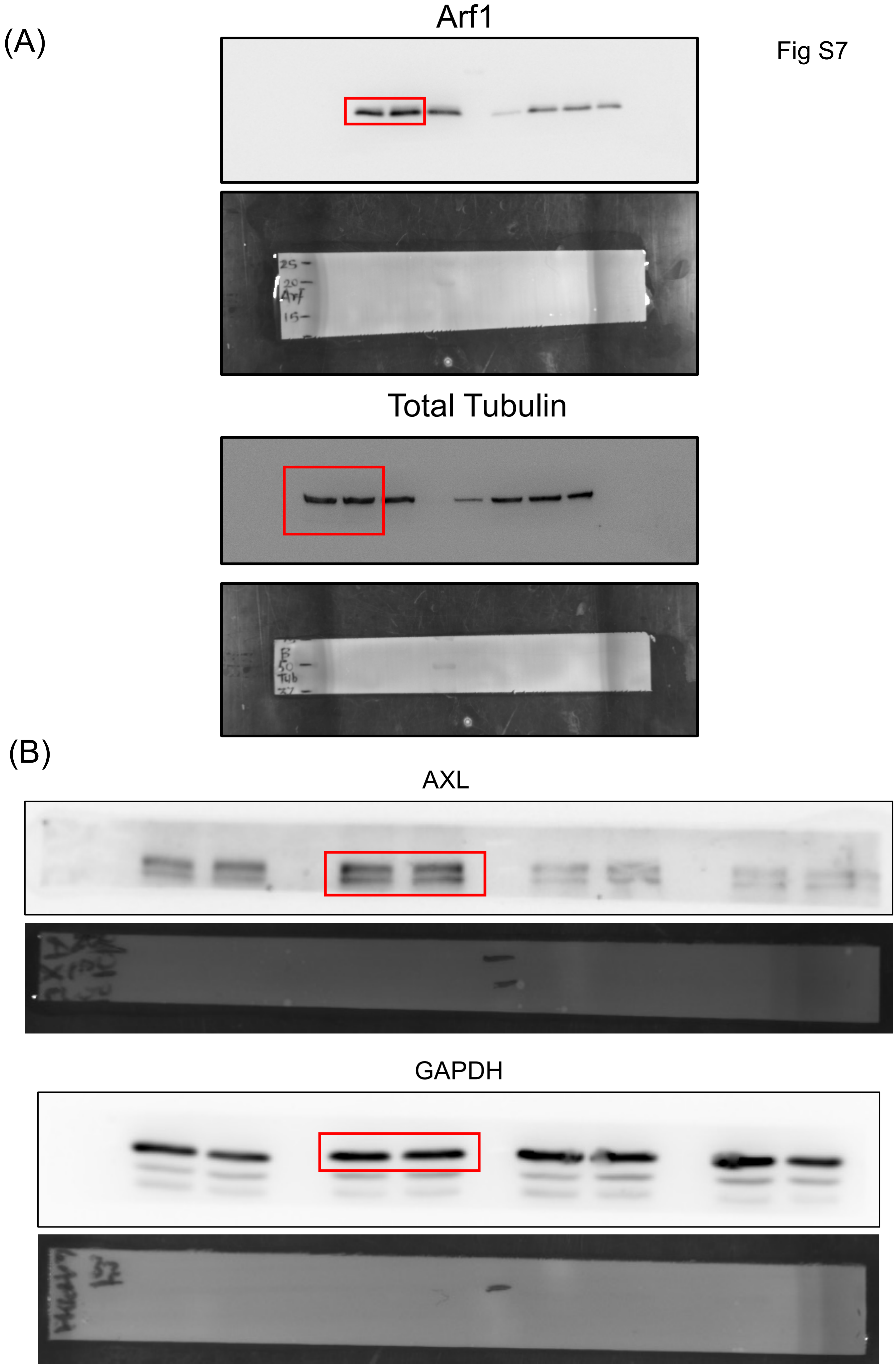

Journal of Cell Science • Supplementary information

Fig S7

(C)

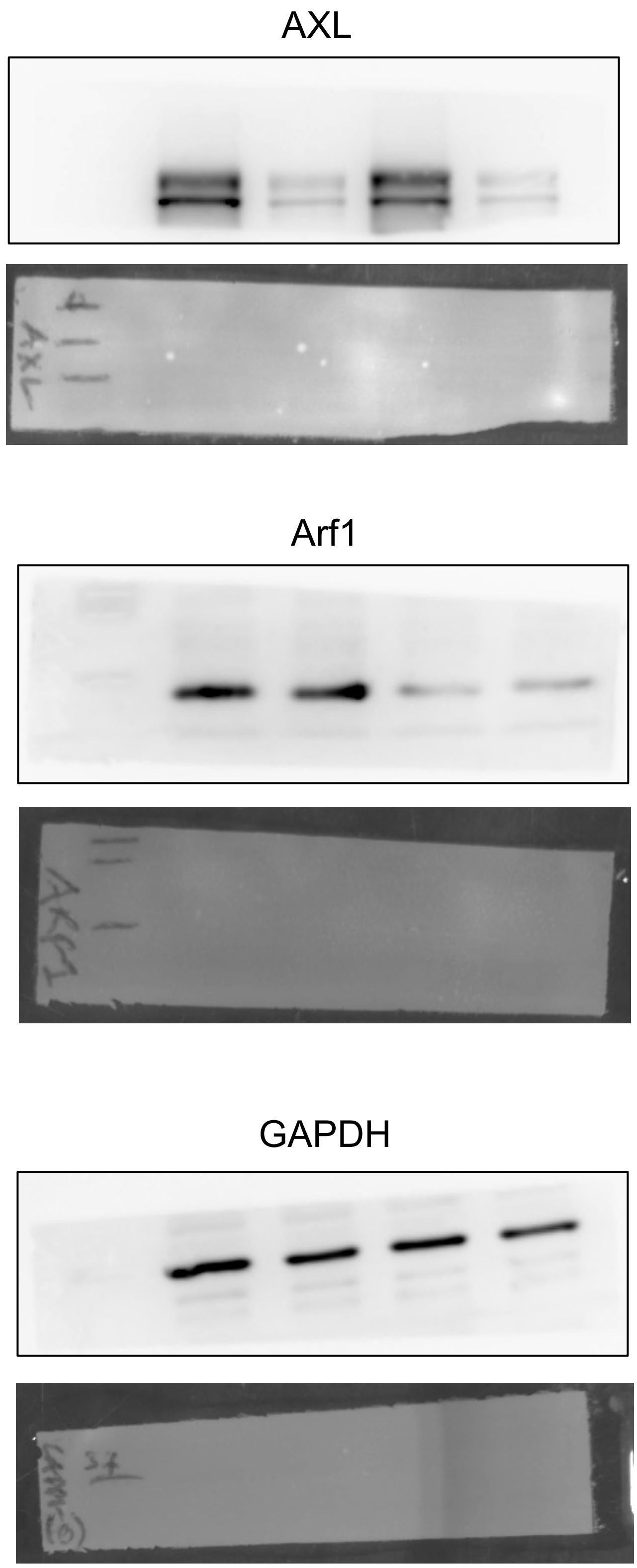

Fig. S8. Blot Transparency.

Journal of Cell Science • Supplementary information
